# TSPAN4 is a prognostic and immune target in Glioblastoma multiforme

**DOI:** 10.3389/fmolb.2022.1030057

**Published:** 2023-01-06

**Authors:** Yue Zheng, Yuheng Lang, Bingcai Qi, Yuchao Wang, Wenqing Gao, Tong Li

**Affiliations:** ^1^ School of Medicine, Nankai University, Tianjin, China; ^2^ Department of Heart Center, The Third Central Hospital of Tianjin, Tianjin, China; ^3^ Nankai University Affiliated Third Center Hospital, Tianjin, China; ^4^ Tianjin Key Laboratory of Extracorporeal Life Support for Critical Diseases, Tianjin, China; ^5^ Artificial Cell Engineering Technology Research Center, Tianjin, China; ^6^ The Third Central Clinical College of Tianjin Medical University, Tianjin, China

**Keywords:** atherosclerosis, GO/KEGG pathways, pan-cancer, GSEA, TSPAN4, macrophages, migrasomes, GBMLGG

## Abstract

**Background:** Atherosclerosis can impact cancer progression due to the cholesterol and calcium metabolism, illustrating the links between atherosclerosis and cancer metastasis. Tetraspanin 4 (TSPAN4) may help understand migrasomes in diseases and provide novel targets for treatment.

**Methods:** TSPAN4 expression in atherosclerosis Gene Expression Omnibus (EO) dataset and multiple omics data were explored, such as enriched pathways analysis, protein-protein interaction analysis, immune subtypes as well as diagnostic and prognostic value in pan-cancer. The relationship between Glioblastoma multiforme (GBM) and TSPAN4 was further investigated.

**Results:** Compared to control, TSPAN4 expression was upregulated in foam cells from patients with atherosclerosis and survival analysis demonstrated high TSPAN4 expression contributes to poor prognosis. TSPAN4 expression differs significantly in immune subtypes of cancers, which can be a diagnostic and prognostic target of cancers due to the high accuracy. Overall survival analysis of subgroups demonstrated that higher TSPAN4 expression had a worse prognosis and the univariate analysis and multivariate analysis demonstrated age, TSPAN4 expression, WHO grade, IDH status and histological types were independent risk factors of Glioblastoma multiforme.

**Conclusion:** The TSPAN4 expression was associated with atherosclerosis progression and pan-cancer, especially in Glioblastoma multiforme and GBMLGG. Therefore, TSPAN4 may serve as a potential biomarker and the crosstalk between atherosclerosis and tumor progression. The results are not fully validated and further studies are still needed to validate *in vivo* and *in vitro*.

## 1 Introduction

Coronary artery disease (CAD) contributes to considerable mortality and morbidity, leading to over one in every seven deaths all over the world (Naghavi et al., 2017). The mortality of patients with atherosclerosis and acute myocardial infarction (MI) has increased 5.6-fold in the last 3 decades and obesity has become the major cause in patients with some chronic diseases, for instance, diabetes and CAD (Chang et al., 2017; Singh et al., 2020). Young patients with type 2 diabetes and MI develop higher long-term cardiovascular-related mortality and more than one-third die within 10 years (Dutta et al., 2012; Chang et al., 2017; Singh et al., 2020).

Tetraspanin 4 (TSPAN4), as a required protein for migrasome formation (Jiang et al., 2019), can interact with the histamine H4 receptor (H4R) in transfected cells (Ma et al., 2021). Previous reports demonstrated that podocytes release the “injury-related” migrasomes (Liu et al., 2020) and migrasome formation is mediated by the assembly of micron-scale tetraspanin macrodomains, for instance, TSPAN4 (Huang et al., 2019). Therefore, the investigation of TSPAN4 functions may help understand migrasomes in diseases and provide novel targets for treatment.

Aseptic inflammation can promote neutrophil extracellular traps (NETs) increase in the liver, thus promoting cancer metastasis (Yang et al., 2020). Graeme et al. also reported MI can epigenetically reprogram Ly6C^high^ monocytes in the bone marrow, which were increasingly recruited to the breast cancer microenvironment and promoted MI-induced early-stage breast cancer progression and increased breast cancer patients’ mortality and morbidity (Koelwyn et al., 2020). Atherosclerosis can impact cancer progression due to the cholesterol and calcium metabolism, illustrating the links between atherosclerosis and cancer metastasis (DUNGAL and BENEDIKTSSON, 1958; Tapia-Vieyra et al., 2017; Balzan and Lubrano, 2018; Mensah et al., 2021). Therefore, the investigation between CAD, especially MI and atherosclerosis, and tumor progression may provide potential biomarkers to impede tumor progression, thus reducing mortality and morbidity. However, the researches on such crosstalk are little.

In this study, the differentially expressed genes (DEGs) were investigated in foam cells from subjects with atherosclerosis in GSE9874 and enrichment pathways were applied to explore macrophage-related DEGs. Next, we determined the expression of TSPAN4 in atherosclerosis and pan-cancer and the prognostic and diagnostic value of TSPAN4 in pan-cancer, and the correlation between the TSPAN4 expression and immune cells were evaluated as well. DEGs between TSPAN4 high and low expression groups were also explored to validate whether TSPAN4 can be the crosstalk between MI and cancer progression. After that, the subgroup analysis, survival analysis and prognostic value of Glioblastoma multiforme and Brain Lower Grade Glioma (GBMLGG) were determined. Finally, the validation of TSPAN4 expression in macrophages was also carried out.

## 2 Materials and methods

### 2.1 Data source and processing

Using the keywords “atherosclerosis” in “*Homo sapiens*,” GSE9874 from the Gene Expression Omnibus (GEO) database was investigated, processed with log2 transformation for normalization and analyzed using the limma package in R (Hägg et al., 2008). The RNA-sequencing of macrophages was based on the Affymetrix Human Genome U133A Array.

The Cancer Genome Atlas (TCGA) database and the Genotype-Tissue Expression (GTEx) database by UCSC XENA (https://portal.gdc.cancer.gov/) were investigated. The gene expression data TCGA pan-cancer, including unpaired samples and paired samples, were analyzed using Xiantao website tool (www.xiantao.love) and GEPIA (cancer-pku.cn) (Tang et al., 2017).

The single-cell sequencing data about TSPAN4 expression in GBMLGG were used from Single Cell Portal (Study: BTSC dependence on GLS reveals a metabolic vulnerability, https://singlecell.broadinstitute.org/) (Restall et al., 2020).

### 2.2 Enrichment pathways analysis

GO/KEGG pathways were explored among macrophage-related DEGs between patients with subclinical atherosclerosis and control (Ashburner et al., 2000; Kanehisa and Goto, 2000; Subramanian et al., 2005a; Yu et al., 2012). A *p*-value < .05 were regarded as the cut-off criterion.

### 2.3 Prognostic value of TSPAN4 expression

Overall survival (OS), Disease Specific Survival (DSS) and Progress Free Interval (PFI) were explored in pan-cancer. In addition, the TSPAN4 expression was also investigated in GBMLGG, Colon adenocarcinoma and Rectum adenocarcinoma Esophageal carcinoma (COADREAD) and Lung adenocarcinoma and Lung squamous cell carcinoma (LUADLUSC).

### 2.4 Diagnostic value of TSPAN4 expression

The receiver operation characteristic (ROC) curve was conducted to investigate the diagnostic performance of TSPAN4 expression and the area under the curve (AUC) was determined using the “pROC” package.

### 2.5 TSPAN4 expression association with immune cells

ssGSEA (GSVA package in R) was used to provide a critical assessment of the relationships between TSPAN4 expression and macrophages in TCGA data (Subramanian et al., 2005b; Yu et al., 2012). A *p*-value < .05 were regarded as significant.

### 2.6 DEGs between TSPAN4 high and low expression groups

The differentially expressed genes (DEGs) between different TSPAN4 expression groups (low expression group: 0%–50%; high expression group: 50%–100%) in LUADLUSC, PRAD, THYM and GBMLGG. A log2(Fold Change) > 1 and a *p*-value < .05 were applied as the cut-off criteria of PRAD and a log2(Fold Change) > 2 and a *p*-value < .05 were applied as the cut-off criteria of LUADLUSC, THYM and GBMLGG.

### 2.7 Analysis of MMR in cancer

The correlation between TSPAN4 expression and several essential mismatch repair (MMR) genes was investigated in LUSC, LUADLUSC, PRAD, THYM, GBM and GBMLGG, including the MutL homologous gene (MLH1), MutS homologous gene (MSH2, MSH6), increased separation after meiosis (PMS2) and epithelial cell adhesion molecule (EPCAM).

### 2.8 Subgroup analysis and survival analysis in GBMLGG

To validate the potential effects of TSPAN4 expressions on GBMLGG progression, the TSPAN4 expressions in subgroups were determined and OS analysis of subgroups was also carried out. Cox regression analysis was used to explore the prognosis of ARL6IP5 expression in subgroups.

### 2.9 Co-expression gene analysis of TSPAN4 expression in GBMLGG

The top 50 co-expression genes positively and negatively related to TSPAN4 expression in GBMLGG were explored. GO/KEGG pathways analysis was used to investigate the related enriched pathways.

### 2.10 Protein-protein interaction (PPI) and the hub genes

To investigate TSPAN4 and its protein interactions, STRING database (https://string-db.org) was used with a combined score >.4 (Szklarczyk et al., 2019). The nodes were analyzed using Cytoscape v.3.7.1 (Shannon et al., 2003). PPI network analysis was used to obtain the hub genes utilizing the Cytoscape plug-in MCODE. The Cytoscape plug-in cytohubba was also used to obtain the genes in top15 MCC, top15 DMNC and top15 Degree.

### 2.11 Prognostic value of TSPAN4 expression in GBM

The nomogram, Calibration curves and time-dependent ROC analysis were used. Lasso regression and risk score were also utilized to explore the functions of TSPAN4 expression on GBM.

### 2.12 Validation of TSPAN4 expression in macrophages

To further validate the TSPAN4 effects, the atherosclerosis mice model and macrophage cell lines were utilized. RAW264.7 and THP-1 macrophage cell lines were purchased from ATCC and adult experimental ApoE^−/−^ male mice were purchased from Charles River. Mice were maintained in an SPF environment (temperature: 23°C–25°C; humidity: 55%–60%) with free access to food and water and a 12/12 light-dark cycle. Protocols were approved by the Institute of Radiation Medicine, the Chinese Academy of Medical Science, which conform to the Guide for the Care and Use of Laboratory Animals.

To construct the atherosclerosis model, the ApoE^−/−^ male mice (*n* = 6 per group) were fed with a chow diet or a western diet (HFHC100244) for 3 months or 6 months. In the atherosclerosis mice model, the aorta root was collected and qRT-PCR and ELISA were performed. In macrophages hypoxia cell model, the RAW264.7 and THP-1 macrophages were cultured in DMEM (Gibco) supplemented with 10% fetal bovine serum (FBS, Auxgen) and treated in a hypoxia chamber filled with 5% CO_2_, 94% N_2_ and 1% O_2_ at 37.0°C for 12 h or 24 h. Then the Tspan4 mRNA level was determined using qRT-PCR and the secreted TSPAN4 protein expression was evaluated using ELISA in a cultured medium.

Total RNA was collected using TRIzol and RNA purity was tested. Total RNA was used for reverse transcription reaction and the amplified cDNA samples were mixed using TB Green^®^ Premix Ex Taq™ II (TaKaRa, RR820). Finally, the reaction was conducted on AriaMx HRM. β-actin was used as a positive control and the 2^−ΔΔCT^ method was used to calculate each sample. Tspan4: F: TAC​CTC​ATG​TTC​GCC​TTC​AAC; R: GAT​AAG​GTG​GCA​AAG​TTT​CCC​T. β-actin: F: GGC​TGT​ATT​CCC​CTC​CAT​CG; R: CCA​GTT​GGT​AAC​AAT​GCC​ATG​T.

ELISA was performed. The TSPAN4 protein in the aorta root was extracted according to the Kit Instruction (BC3710, Solarbio). For further validation, the TSPAN4 protein levels in the aorta root and macrophages cultured medium were explored with the 4°C incubation overnight of mouse TSPAN4 primary antibodies (1:600, LS-C676657-50, LSBio). The serum matrix (MILLIPLEX Analyst Kit, Millipore) was used as the positive control in the validation.

### 2.13 Statistical analysis

All data are presented as the mean ± SD. Statistical analyses were performed using SPSS 23.0. Shapiro-Wilk normality test and Wilcoxon rank sum test were used and a value of *p* < .05 was considered statistically significant.

## 3 Results

### 3.1 TSPAN4 was highly expressed in atherosclerosis

TSPAN4 was highly expressed in foam cells from the patients with atherosclerosis, while there was no significant difference in TSPAN4 expression in foam cells from the subjects without atherosclerosis compared to baseline (Supplementary Figure S1). GO/KEGG pathways of DEGs in atherosclerosis were also applied, which were mainly involved in regulation of lipid metabolic process, monooxygenase activity, and NF-kappa B signaling pathway (Supplementary Figure S2; Supplementary Tables S1, S2).

### 3.2 TSPAN4 was highly expressed in pan-cancer

**FIGURE 1:**
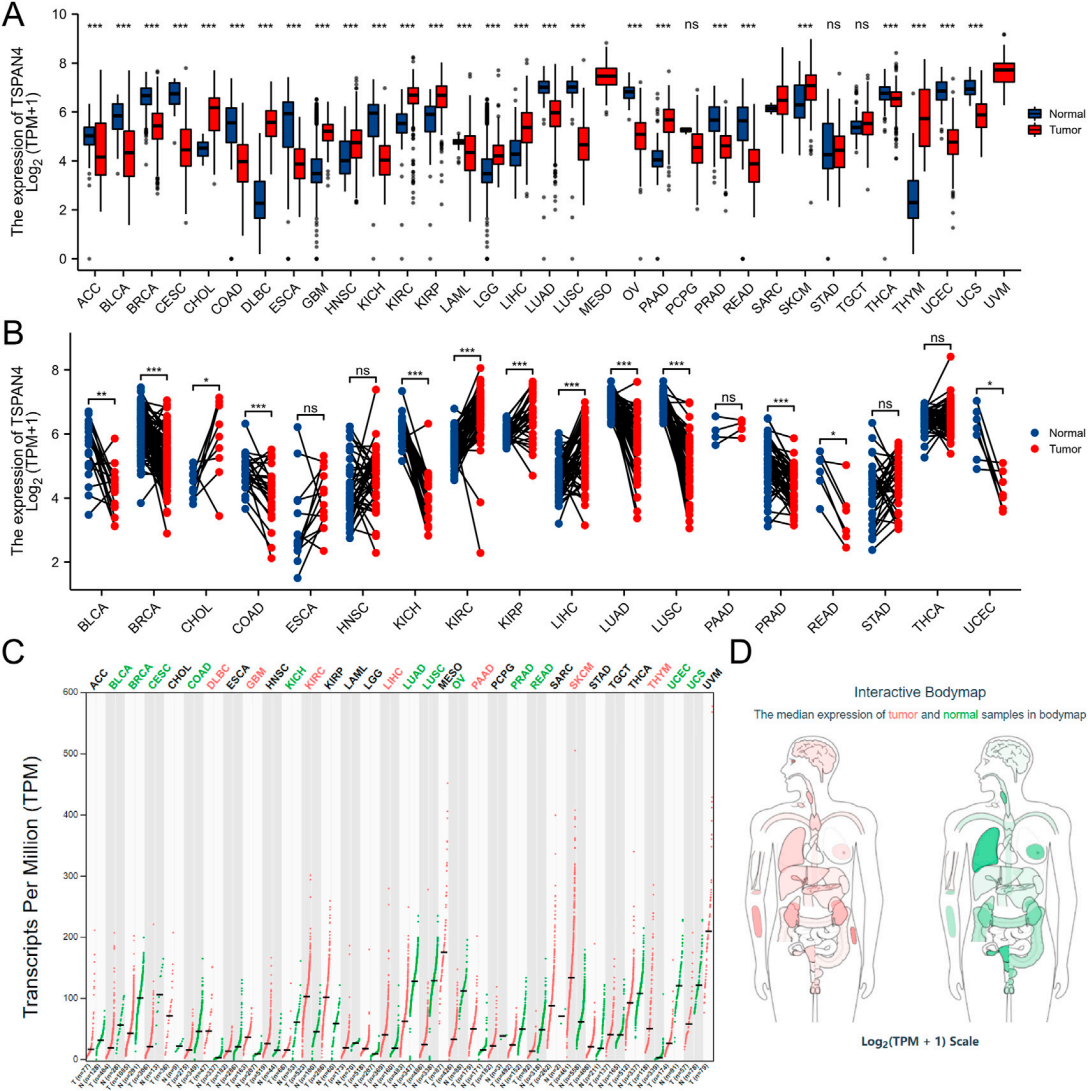
TSPAN4 expression in pan-cancer. **(A)** Unpaired samples in pan-cancer demonstrated there were significant differences of TSPAN4 expression in 27 cell lines in TCGA. **(B)** Paired samples in pan-cancer demonstrated there were significant differences of TSPAN4 expression in 13 cell lines in TCGA. **(C)** Using GEPIA, TSPAN4 was highly expressed in 7 tumor cell lines and lowly expressed in 12 tumor cell lines. **(D)** The median expression of tumor and normal samples in bodymap. Red, tumor samples; green, normal samples.

The gene expression data in TCGA pan-cancer were extracted, filtered and analyzed (Figure 1). Unpaired samples in pan-cancer demonstrated there were significant differences in TSPAN4 expression in 27 cell lines in TCGA, including Adrenocortical carcinoma (ACC), Bladder Urothelial Carcinoma (BLCA), Breast invasive carcinoma (BRCA), Cervical squamous cell carcinoma and endocervical adenocarcinoma (CESC), Cholangiocarcinoma (CHOL), Colon adenocarcinoma (COAD), Lymphoid Neoplasm Diffuse Large B-cell Lymphoma (DLBC), Esophageal carcinoma (ESCA), GBM, Head and Neck squamous cell carcinoma (HNSC), Kidney Chromophobe (KICH), Kidney renal clear cell carcinoma (KIRC), Kidney renal papillary cell carcinoma (KIRP), Acute Myeloid Leukemia (LAML), Brain Lower Grade Glioma (LGG), Liver hepatocellular carcinoma (LIHC), Lung adenocarcinoma (LUAD), LUSC, Ovarian serous cystadenocarcinoma (OV), Pancreatic adenocarcinoma (PAAD), PRAD, Rectum adenocarcinoma (READ), Skin Cutaneous Melanoma (SKCM), Thyroid carcinoma (THCA), THYM, Uterine Corpus Endometrial Carcinoma (UCEC) and Uveal Melanoma (UCS). Paired samples in pan-cancer demonstrated there were significant differences in TSPAN4 expression in 13 cell lines in TCGA, including BLCA, BRCA, CHOL, COAD, KICH, KIRP, LIHC, LUAD, LUSC, PRAD, READ and UCEC. Using GEPIA, TSPAN4 was highly expressed in 7 tumor cell lines, including DLBC, GBM, KIRC, LIHC, PAAD, SKCM and THYM, and lowly expressed in 12 tumor cell lines, including BLCA, BRCA, CESC, COAD, KICH, LUAD, LUSC, OV, PRAD, READ, UCEC and UCS.

Pan-cancer analysis was also used in TSPAN7 expression (Supplementary Figure S3). Unpaired samples in pan-cancer demonstrated there were significant differences in TSPAN7 expression in 17 cell lines in TCGA. Paired samples in pan-cancer demonstrated there were significant differences in TSPAN7 expression in 13 cell lines in TCGA.

### 3.3 Prognostic value of TSPAN4 expression in pan-cancer

**FIGURE 2:**
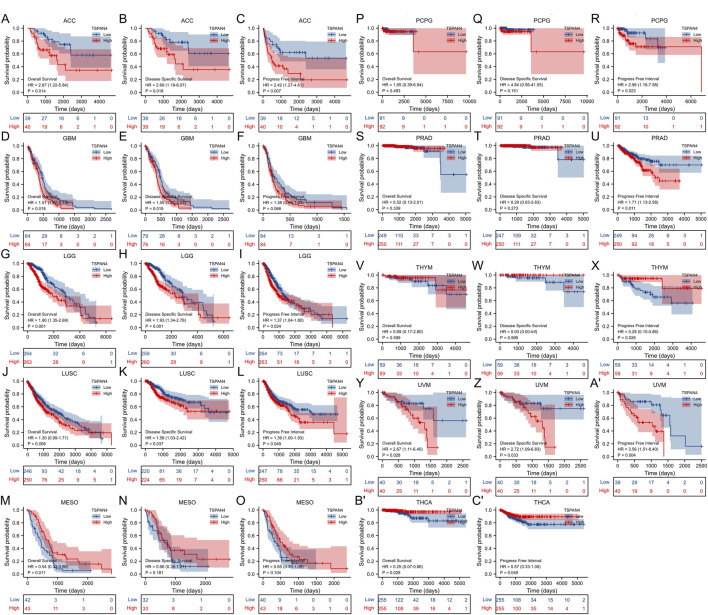
Prognostic value of TSPAN4 expression in pan-cancer. **(A–C)** The overall survival analysis **(A)**, diseases specific survival analysis **(B)** and progress free interval analysis **(C)** of TSPAN4 expression in ACC. **(D–F)** The overall survival analysis **(D)**, diseases specific survival analysis **(E)** and progress free interval analysis **(F)** of TSPAN4 expression in GBM. **(G–I)** The overall survival analysis **(G)**, diseases specific survival analysis **(H)** and progress free interval analysis **(I)** of TSPAN4 expression in LGG. **(J–L)** The overall survival analysis **(J)**, diseases specific survival analysis **(K)** and progress free interval analysis **(L)** of TSPAN4 expression in LUSC. **(M–O)** The overall survival analysis **(M)**, diseases specific survival analysis **(N)** and progress free interval analysis **(O)** of TSPAN4 expression in MESO. **(P–R)** The overall survival analysis **(P)**, diseases specific survival analysis **(Q)** and progress free interval analysis **(R)** of TSPAN4 expression in PCPG. **(S–U)** The overall survival analysis **(S)**, diseases specific survival analysis **(T)** and progress free interval analysis **(U)** of TSPAN4 expression in PRAD. **(V–X)** The overall survival analysis **(V)**, diseases specific survival analysis **(W)** and progress free interval analysis **(X)** of TSPAN4 expression in THYM. **(Y-A′)** The overall survival analysis **(Y)**, diseases specific survival analysis **(Z)** and progress free interval analysis **(A′)** of TSPAN4 expression in UVM. **(B′,C′)** The overall survival analysis **(B′)** and progress free interval analysis **(C′)** of TSPAN4 expression in THCA.

**FIGURE 3:**
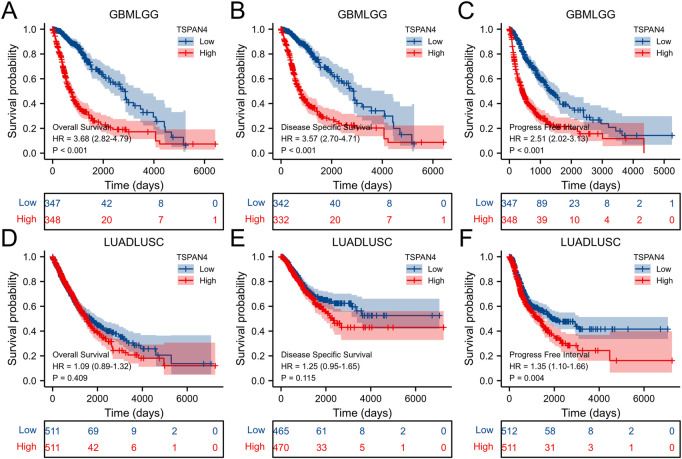
Prognostic value of TSPAN4 expression in GBMLGG and LUADLUSC. **(A–C)** The overall survival analysis **(A)**, diseases specific survival analysis **(B)** and progress free interval analysis **(C)** of TSPAN4 expression in GBMLGG. **(D–F)** The overall survival analysis **(D)**, diseases specific survival analysis **(E)** and progress free interval analysis **(F)** of TSPAN4 expression in LUADLUSC.

The overall survival analysis, diseases specific survival analysis and progress free interval analysis of TSPAN4 expression demonstrated that high TSPAN4 expression was correlated to poor prognosis of the patients with ACC, GBM, LGG, LUSC, MESO, PCPG, PRAD, THYM, UVM, THCA, GBMLGG or LUADLUSC (Figures 2, 3).

### 3.4 Diagnostic value of TSPAN4 expression in pan-cancer

**FIGURE 4:**
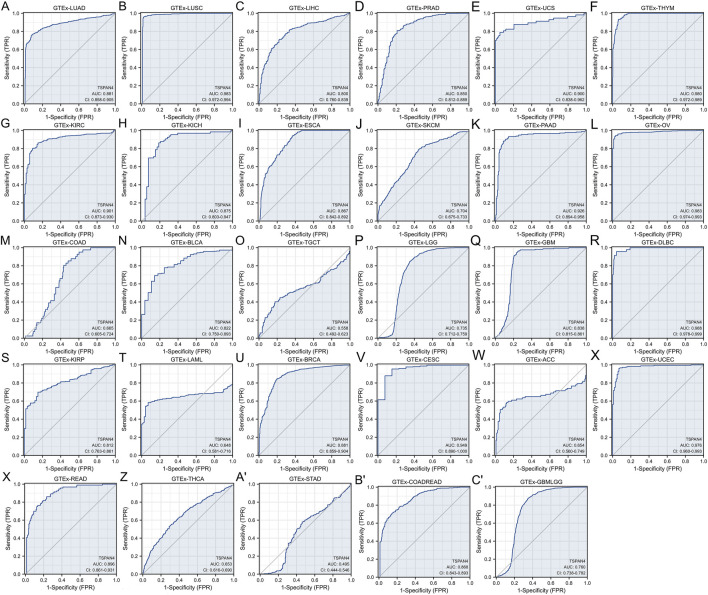
Diagnostic value of TSPAN4 expression in pan-cancer and GTEx, including GTEx-LUAD **(A)**, GTEx-LUSC **(B)**, GTEx-LIHC **(C)**, GTEx-PRAD **(D)**, GTEx-UCS **(E)**, GTEx-THYM **(F)**, GTEx-KIRC **(G)**, GTEx-KICH **(H)**, GTEx-ESCA **(I)**, GTEx-SKCM **(J)**, GTEx-PAAD **(K)**, GTEx-OV **(L)**, GTEx-COAD **(M)**, GTEx-BLCA **(N)**, GTEx-TGCT **(O)**, GTEx-LGG **(P)**, GTEx-GBM **(Q)**, GTEx-DLBC **(R)**, GTEx-KIRP **(S)**, GTEx-LAML **(T)**, GTEx-BRCA **(U)**, GTEx-CESC **(V)**, GTEx-ACC **(W)**, GTEx-UCEC **(X)**, GTEx-READ **(Y)**, GTEx-THCA **(Z)**, GTEx-STAD **(A′)**, GTEx-COADREAD **(B′)** and GTEx-GBMLGG **(C′)**. TSPAN4 expression can diagnose 20 cancer types compared to normal samples (AUC > .8), including LUAD, LUSC, LIHC, PRAD, UCS, THYM, KIRC, KICH, ESCA, PAAD, OV, BLCA, GBM, DLBC, KIRP, BRCA, CESC, UCEC, READ and COADREAD.

To explore the diagnostic value of TSPAN4 expression in pan-cancer, ROC analysis was used, suggesting that the TSPAN4 expression can be credible biomarkers in LUSC, UCS, THYM, KIRC, PAAD, OV, DLBC, CESC and UCEC (AUC > .9), and TSPAN4 expression can also be potential biomarkers in LUAD, LIHC, PRAD, KICH, ESCA, BLCA, GBM, KIRP, BRCA, READ and COADREAD (AUC > .8) (Figure 4).

After evaluation, the TSPAN4 expressions in GBM, LUSC, PRAD and THYM, were further analyzed according to the high prognostic and diagnostic values.

### 3.5 TSPAN4 expression and immune cells analysis

**FIGURE 5:**
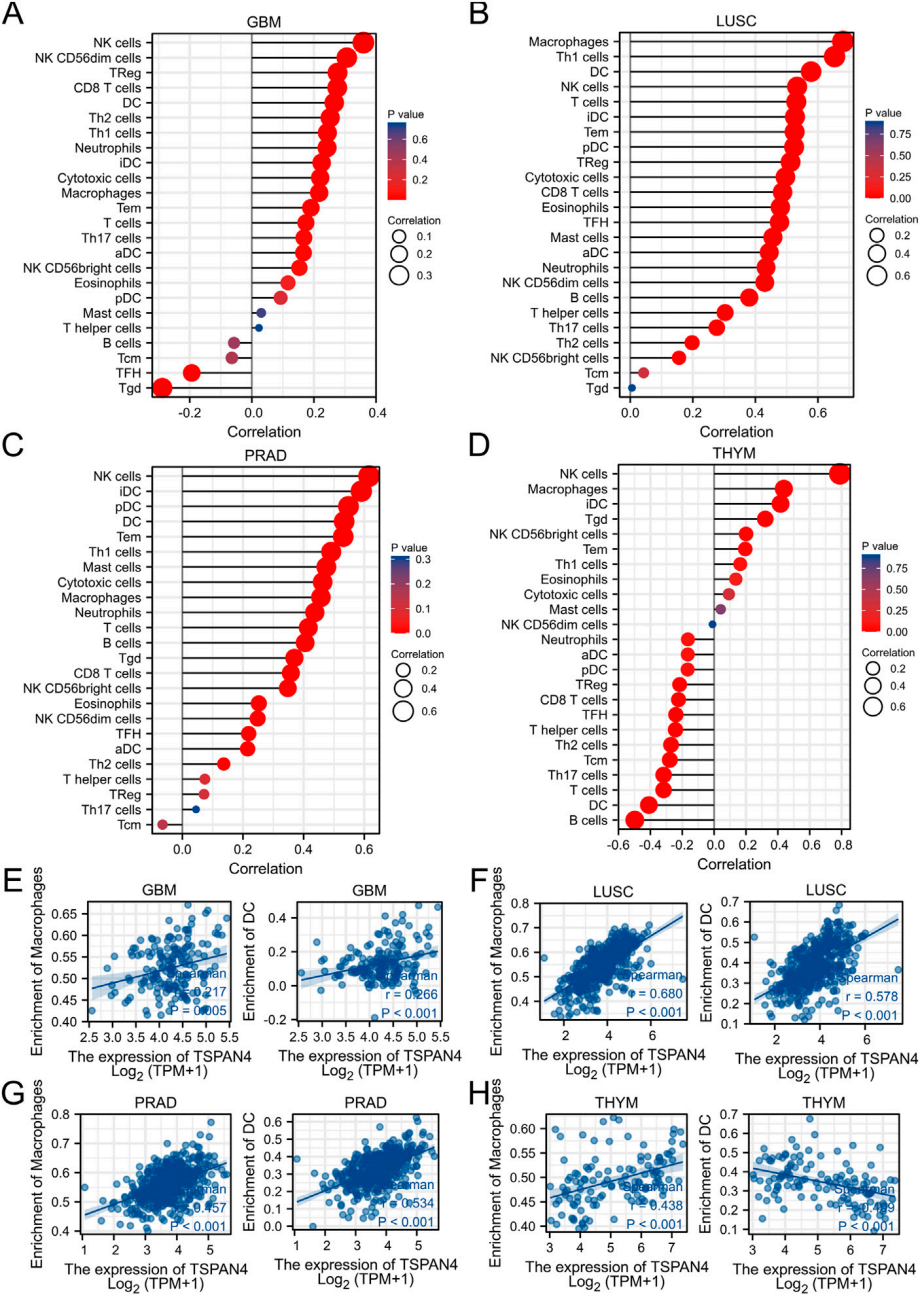
TSPAN4 expression and immune infiltration analysis. **(A–D)** The lollipop chart of 24 immune cells in GBM **(A)**, LUSC **(B)**, PRAD **(C)** and THYM **(D)**. **(E–H)** The correlation analysis between the abundance of macrophages or DC cells and TSPAN4 expression in GBM **(E)**, LUSC **(F)**, PRAD **(G)** and THYM **(H)**.

**FIGURE 6:**
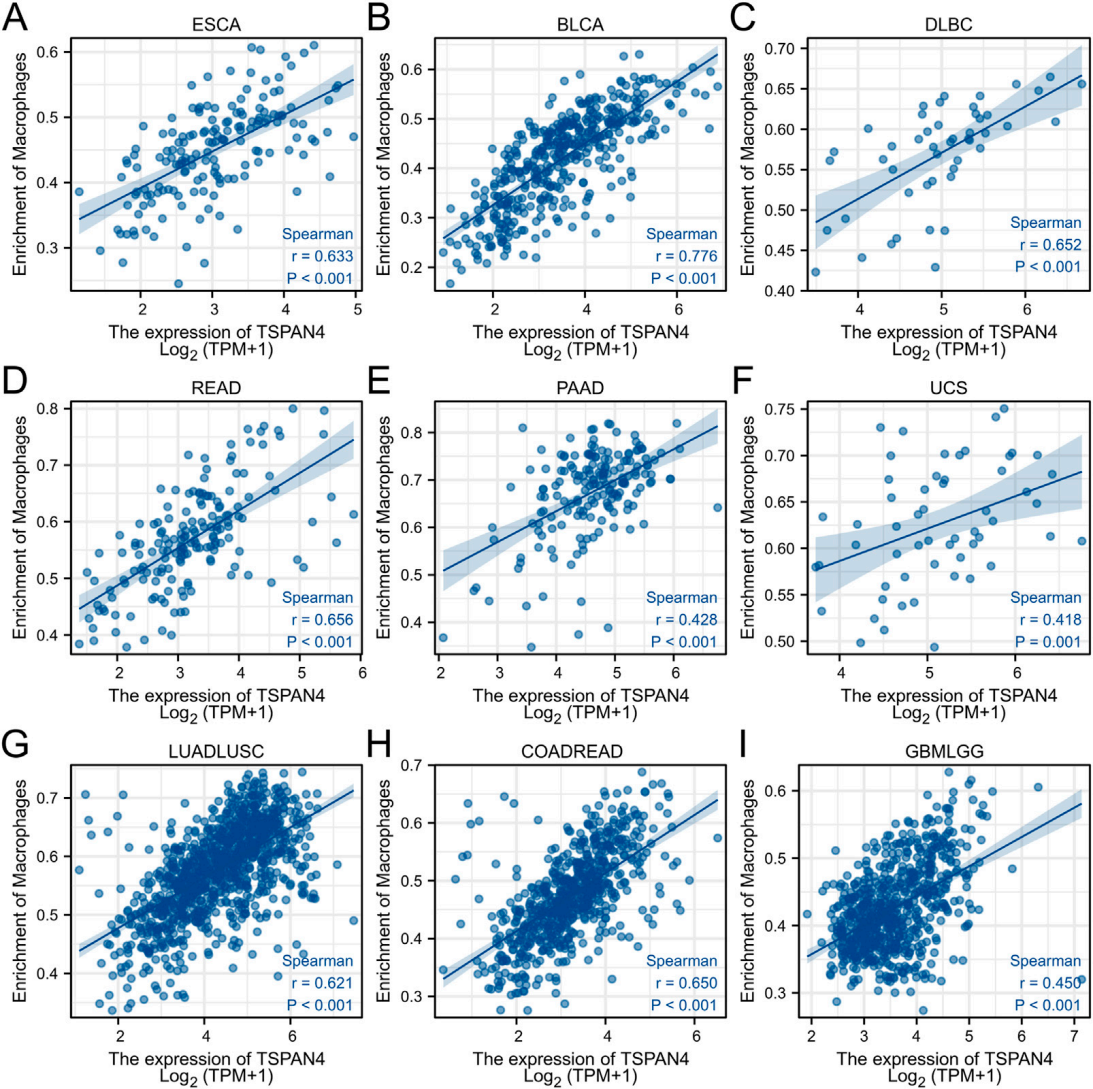
The correlation analysis between the abundance of macrophages and TSPAN4 expression in other cell lines, including ESCA **(A)**, BLCA **(B)**, DLBC **(C)**, READ **(D)**, PAAD **(E)**, UCS **(F)**, LUADLUSC **(G)**, COADREAD **(H)** and GBMLGG **(I)**.

ssGSEA was used in GBM, LUSC, PRAD and THYM, illustrating that TSPAN4 expression was positively correlated to the abundance of macrophages and DC cells in GBM, LUSC and PRAD as well as the abundance of macrophages in THYM. Interestingly, TSPAN4 expression was negatively correlated to the abundance of DC cells (Figure 5). In addition, TSPAN4 expression was highly positively correlated to the abundance of macrophages in ESCA, BLCA, DLBC, READ, PAAD, UCS, LUADLUSC, COADREAD and GBMLGG (Figure 6).

### 3.6 DEGs between TSPAN4 high and low expression groups

**FIGURE 7:**
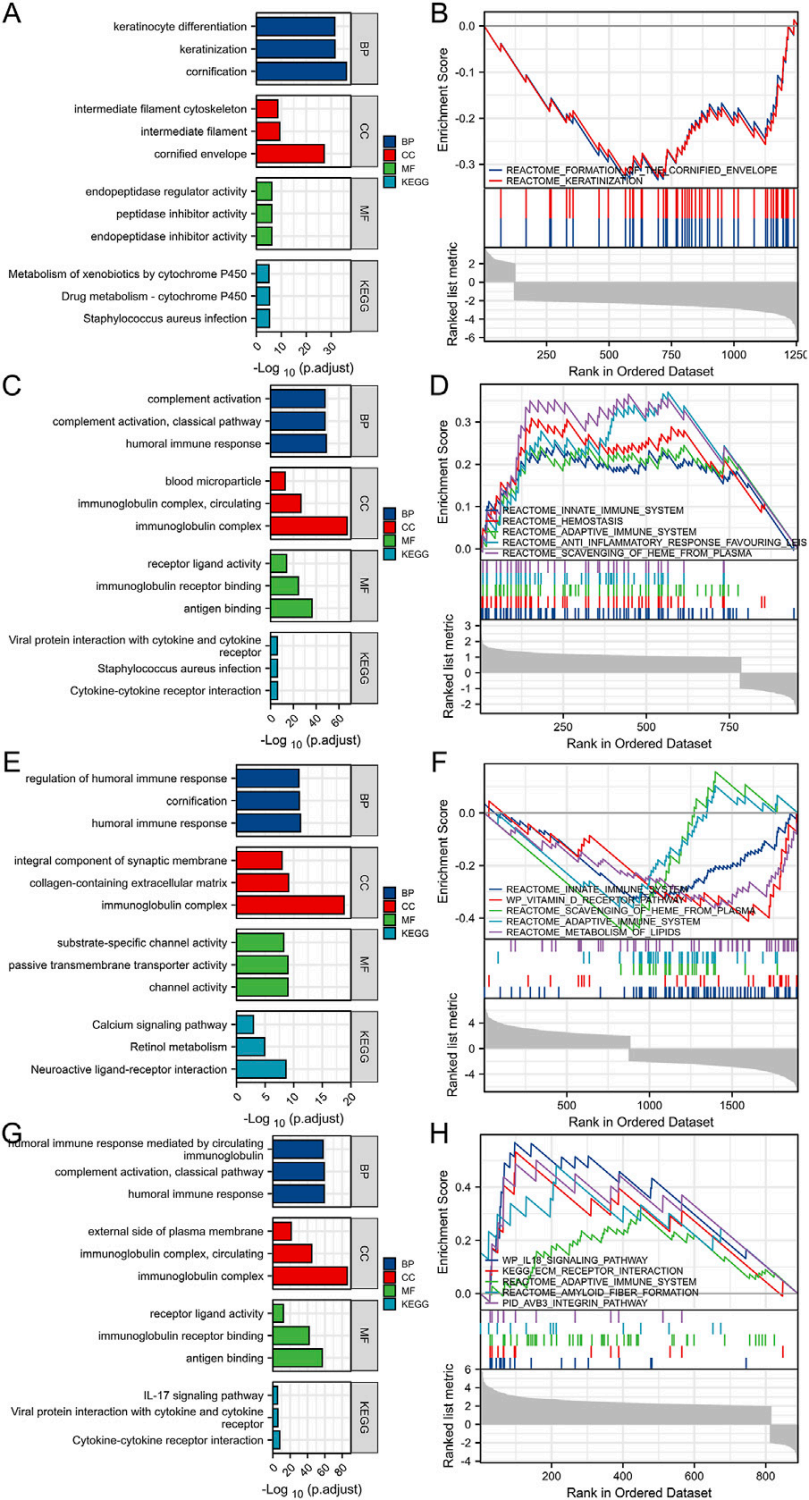
DEGs between TSPAN4 high expression and low expression groups in LUADLUSC, PRAD, THYM and GBMLGG. **(A,B)** The bar plot of GO/KEGG pathways **(A)** and GSEA **(B)** enriched by DEGs between TSPAN4 high expression and low expression groups in LUADLUSC. **(C,D)** The bar plot of GO/KEGG pathways **(C)** and GSEA **(D)** enriched by DEGs between TSPAN4 high expression and low expression groups in PRAD. **(E,F)** The bar plot of GO/KEGG pathways **(E)** and GSEA **(F)** enriched by DEGs between TSPAN4 high expression and low expression groups in THYM. **(G, H)** The bar plot of GO/KEGG pathways **(G)** and GSEA **(H)** enriched by DEGs between TSPAN4 high expression and low expression groups in GBMLGG.

After Log2 transformation, DEGs between TSPAN4 high and low expression groups in LUADLUSC were obtained and further GO/KEGG pathway analysis was applied, which were mainly involved in cornification, keratinocyte differentiation, endopeptidase inhibitor activity, and intermediate filament cytoskeleton. GSEA of DEGs between TSPAN4 high and low expression groups was explored, which were mainly enriched in REACTOME_FORMATION_OF_THE_CORNIFIED_ENVELOPE and REACTOME_KERATINIZATION (Figures 7A, B).

DEGs between TSPAN4 high and low expression groups in PRAD were obtained and further GO/KEGG pathway analysis was applied, which were mainly involved in humoral immune response, protein activation cascade, and receptor ligand activity. GSEA of DEGs between TSPAN4 high and low expression groups was explored, which were mainly enriched in REACTOME_HEMOSTASIS, REACTOME_ANTI_INFLAMMATORY_RESPONSE_FAVOURING_LEISHMANIA_PARASITE_INFECTION, and REACTOME_VESICLE_MEDIATED_TRANSPORT (Figures 7C, D).

DEGs between TSPAN4 high and low expression groups in THYM were obtained and further GO/KEGG pathway analysis was applied, which were mainly involved in regulation of humoral immune response, protein activation cascade, collagen-containing extracellular matrix, intrinsic component of synaptic membrane, and channel activity. GSEA of DEGs between TSPAN4 high and low expression groups was explored, which were mainly enriched in REACTOME_INNATE_IMMUNE_SYSTEM, REACTOME_INITIAL_TRIGGERING_OF_COMPLEMENT, and REACTOME_CD22_MEDIATED_BCR_REGULATION (Figures 7E, F).

DEGs between TSPAN4 high and low expression groups in GBMLGG were obtained and further GO/KEGG pathway analysis was applied, which were mainly involved in immunoglobulin complex, external side of plasma membrane, and antigen binding. GSEA of DEGs between TSPAN4 high and low expression groups was explored, which were mainly enriched in REACTOME_EXTRACELLULAR_MATRIX_ORGANIZATION, WP_IL18_SIGNALING_PATHWAY, and KEGG_ECM_RECEPTOR_INTERACTION (Figures 7G, H).

### 3.7 Analysis of MMR in cancer

**FIGURE 8:**
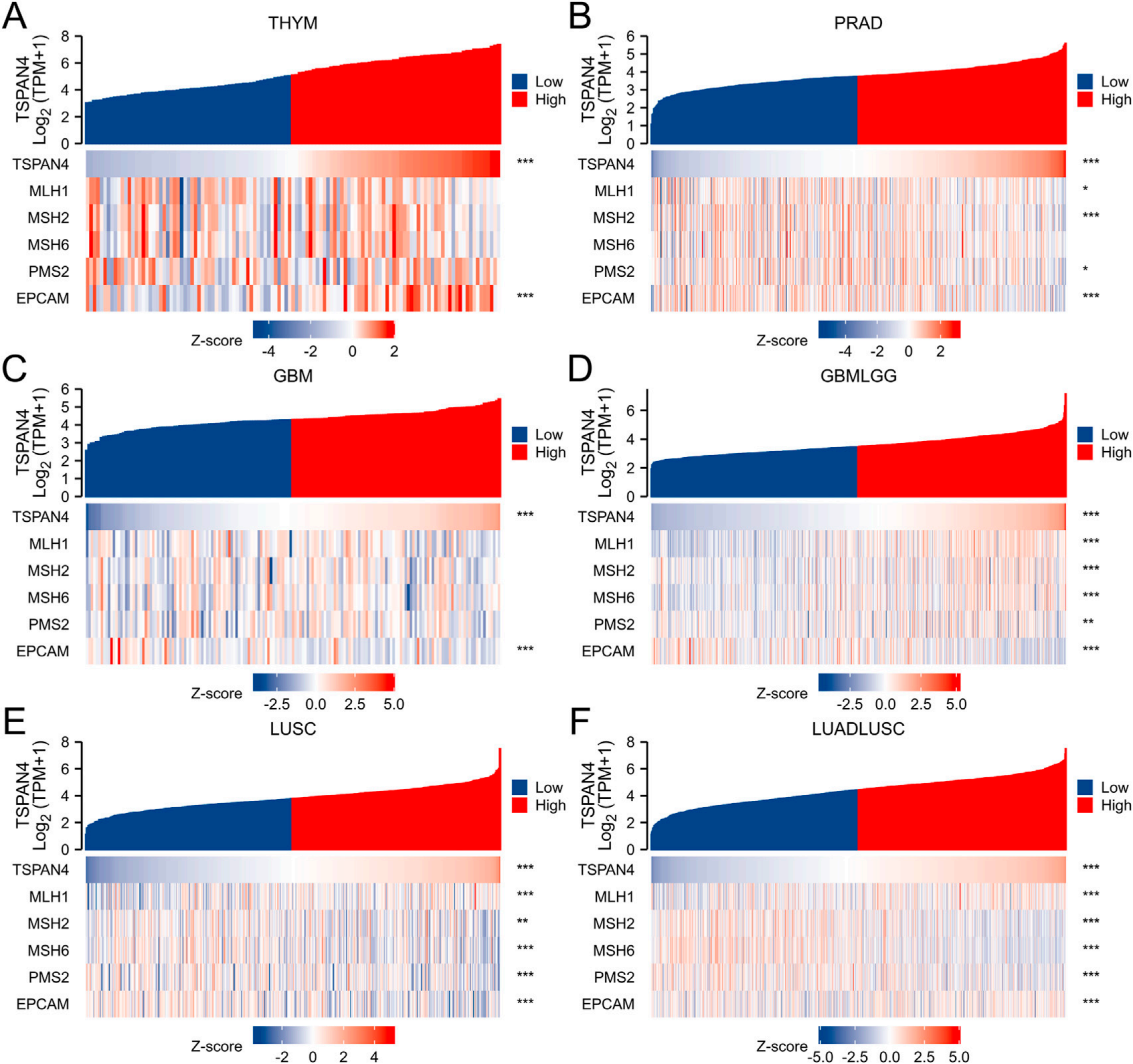
Analysis of MMR in cancer. The correlation between TSPAN4 expression and several essential mismatch repair genes, including MLH1, MSH2, MSH6, PMS2 and EPCAM, was explored in THYM **(A)**, PRAD **(B)**, GBM **(C)**, GBMLGG **(D)**, LUSC **(E)** and LUADLUSC **(F)**.

To investigate the correlation between TSPAN4 expression and several essential mismatch repair genes, including MLH1, MSH2, MSH6, PMS2 and EPCAM, the correlation heatmap of several gene expressions was explored in THYM, PRAD, GBM, GBMLGG, LUSC and LUADLUSC (Figure 8). MLH1, MSH2, MSH6, PMS2 and EPCAM expressions were correlated to TSPAN4 expression in GBMLGG, LUSC and LUADLUSC. MLH1, MSH2, PMS2 and EPCAM expressions were correlated to TSPAN4 expression in PRAD, while only EPCAM expression was correlated to TSPAN4 expression in THYM and GBM.

### 3.8 Subgroup analysis and survival analysis

**FIGURE 9:**
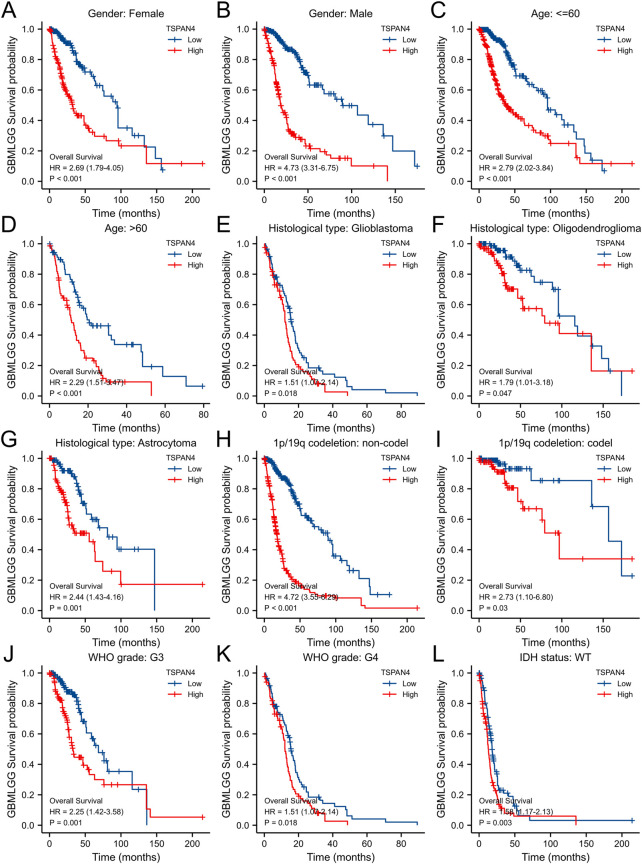
The associations between TSPAN4 expression and the overall survival in different clinical subgroups of GBMLGG. **(A)** Gender: Female; **(B)** Gender: Male; **(C)** Age: ≤60; **(D)** Age: >60; **(E)** Histological type: Glioblastoma; **(F)** Histological type: Oligodendroglioma; **(G)** Histological type: Astrocytoma; **(H)** 1p/19q codeletion: non-codel; **(I)** 1p/19q codeletion: codel; **(J)** WHO grade: G3; **(K)** WHO grade: G4; and **(L)** IDH status: WT.

To validate the potential effects of TSPAN4 expression in GBMLGG, the TSPAN4 expressions in subgroups were determined (Figure 9). Using survival analysis, high TSPAN4 expression contributed to a worse prognosis in OS of GBMLGG subgroup patients, such as patients with Glioblastoma, Oligodendroglioma, Astrocytoma, WHO grade: G3, or WHO grade: G4.

### 3.9 Co-expression gene analysis of TSPAN4 expression in GBMLGG

**FIGURE 10:**
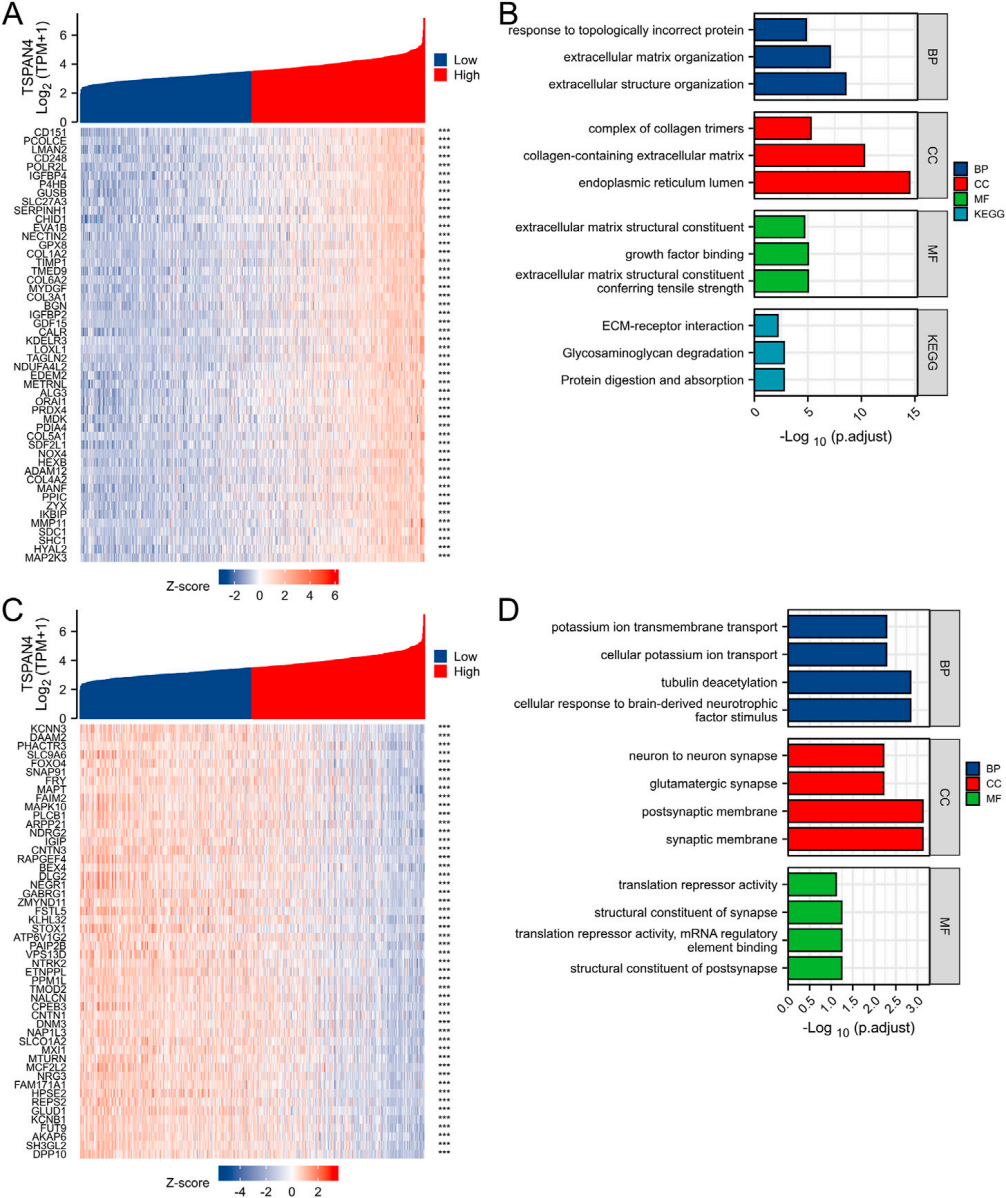
Top 50 genes positively and negatively correlated with TSPAN4 expression in GBMLGG. **(A)** The gene co-expression heatmap of the top 50 genes positively correlated with TSPAN4 expression in GBMLGG. **(B)** The bar plot of GO/KEGG pathways enriched by the top 50 genes positively correlated with TSPAN4 expression. **(C)** The gene co-expression heatmap of the top 50 genes negatively correlated with TSPAN4 expression in GBMLGG. **(D)** The bar plot of GO/KEGG pathways enriched by the top 50 genes negatively correlated with TSPAN4 expression.

The top 50 co-expression genes positively related to TSPAN4 expression in GBMLGG were explored, which were mainly involved in extracellular structure organization, endoplasmic reticulum lumen, collagen-containing extracellular matrix, and growth factor binding (Figures 10A, B; Supplementary Figure S4). The top 50 co-expression genes negatively related to TSPAN4 expression in GBMLGG were also investigated, which were mainly involved in cellular potassium ion transport, synaptic membrane, postsynaptic membrane, and glutamatergic synapse (Figures 10C, D; Supplementary Table S5).

### 3.10 PPI network and the hub genes

To investigate TSPAN4 and its protein interactions in GBMLGG, the nodes with a combined score >.4 were analyzed using STRING. The hub genes were obtained utilizing the Cytoscape plug-in MCODE and there were 4 modules in the network (Supplementary Figure S4). Venn diagram of top15 MCC, DMNC and degree demonstrated several targets, including CXCL11, LOX, LUM, CCL20, VEGFA and COL6A3, can be potential targets for GBMLGG progression (Supplementary Figure S5).

### 3.11 Prognostic value of TSPAN4 expression in GBM

**FIGURE 11:**
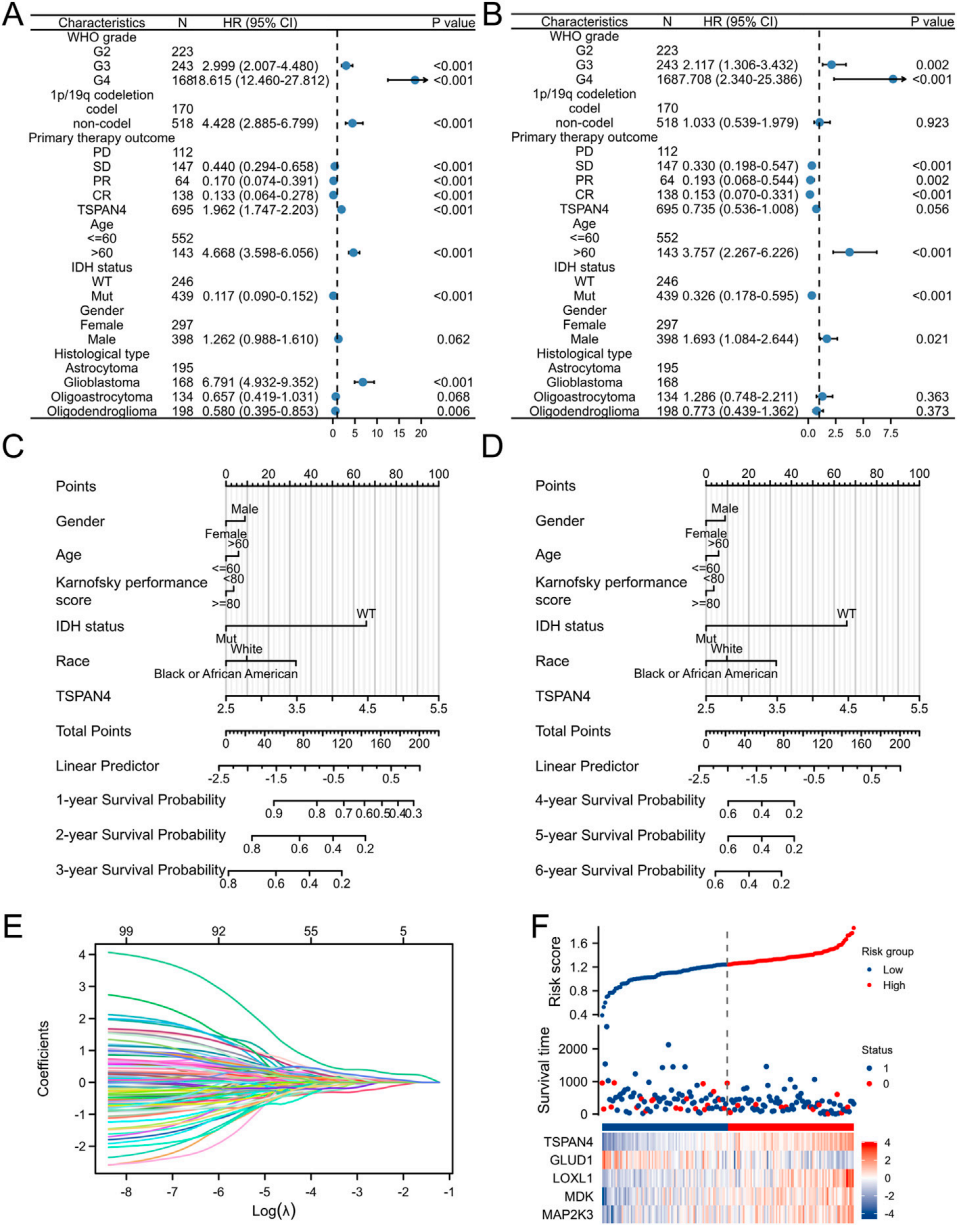
Prognostic value of TSPAN4 expression in GBM. **(A,B)** The forest diagrams of univariate analysis **(A)** and multivariate analysis **(B)** of patients’ characteristics. **(C)** The nomogram about 1-, 2- and 3-year survival probability of TSPAN4 expression and other clinical characteristics in GBM. **(D)** The nomogram about 4-, 5- and 6-year survival probability of TSPAN4 expression and other clinical characteristics in GBM. **(E,F)** The lasso regression **(E)** and risk score analysis **(F)** of survival time and TSPAN4 expression in GBM. 1, survival; 0, dead.

To further investigate the prognostic value of TSPAN4 expression in GBM, the univariate analysis and multivariate analysis of patients’ characteristics demonstrated age, TSPAN4 expression, WHO grade, IDH status and histological types were independent risk factors of GBM (Figures 11A, B; Supplementary Tables S6, S7). To investigate the application of TSPAN4 expression in GBM progression, a nomogram in 1- to 6-year survival probability was constructed and gender, age, TSPAN4 expression and IDH status were included as prognostic factors in the nomogram (Figures 11C, D). The calibration curve suggested that the nomogram was credible in predicting possibility (Supplementary Figures S6A, B). The risk score analysis of lasso regression also illustrated that TSPAN4 expression in GBM was positively associated with risk scores and both were negatively related to survival time (Figures 11E, F; Supplementary Figure S6C). Time-dependent ROC of TAPAN4 expression in GBM and GBMLGG demonstrated that TSPAN4 can be a credible prognostic biomarker in GBM and GBMLGG (Supplementary Figure S7).

### 3.12 Single-cell sequencing of TSPAN4 expression in GBM

**FIGURE 12:**
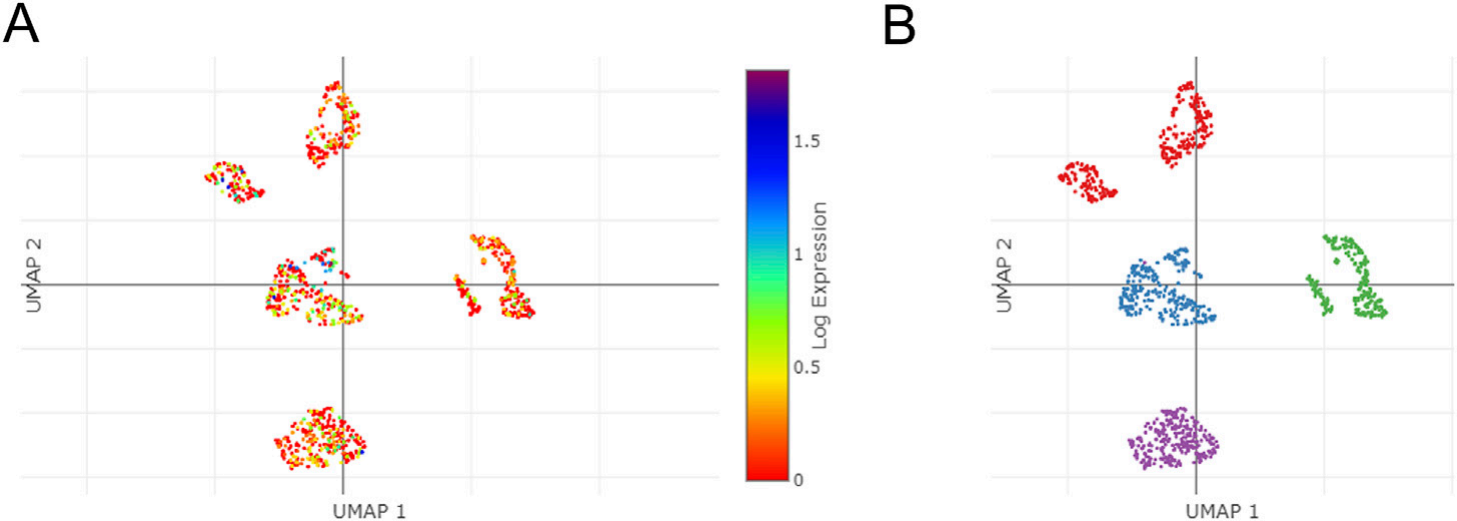
The single cells sequencing of TSPAN4 expression in normal and GBM patients. **(A,B)** TSPAN4 expression **(A)** and the overall **(B)** in sequencing cells in GBM patients using Single Cell Portal database.

Using the single Cell Portal, single-cell sequencing of TSPAN4 expression in GBM was also investigated, suggesting TSPAN4 was highly expressed in all GBM cell types compared to control groups (Figure 12).

### 3.13 Validation using mice model and macrophages cell lines

**FIGURE 13:**
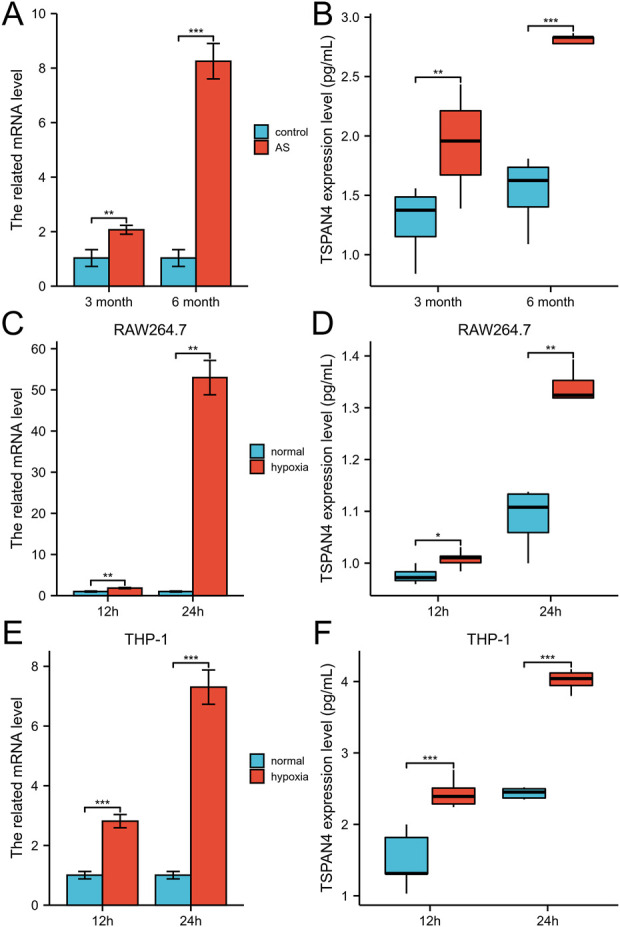
The validation of TSPAN4 effects on atherosclerosis and macrophages. **(A,B)** The related mRNA level **(A)** and ELISA result **(B)** of TSPAN4 expression in ApoE^−/−^ mice fed with high fat diet for 3 months and 6 months. **(C,D)** The related mRNA level **(C)** and ELISA result **(D)** of TSPAN4 expression in RAW264.7 macrophages under hypoxia. **(E,F)** The related mRNA level **(E)** and ELISA result **(F)** of TSPAN4 expression in THP-1 macrophages under hypoxia. AS, atherosclerosis. *p < .05; **p < .01; ***p < .001.

To investigate the TSPAN4 effects on atherosclerosis, the qPCR and ELISA of aorta root in ApoE^−/−^ mice were utilized. The TSPAN4 mRNA and protein levels of the aorta root were highly expressed in atherosclerosis (Figures 13A, B). To explore the TSPAN4 effects on atherosclerosis and GBM progression, RAW264.7 and THP-1 macrophage hypoxia cell lines were utilized. The TSPAN4 mRNA and secreted protein levels were also highly expressed (Figures 13C–F), which may be a potential target for migrasomes and atherosclerosis and GBM progression.

## 4 Discussion

Monocytes and monocyte-derived cells, including macrophages, differ in different phenotypic states from their surrounding microenvironment, which drives T cells activation and function (Padgett et al., 2020). NETs towards atherosclerosis and thrombosis promote breast cancer metastasis to the liver (Moschonas and Tselepis, 2019; Yang et al., 2020). In addition, chemotherapy to treat cancer also induces atherosclerosis plaque formation and progression (Mukai et al., 2018). Therefore, the crosstalk between atherosclerosis and cancer progression needs to be investigated. In this study, TSPAN4 expression in atherosclerosis and pan-cancer was explored and the diagnostic and prognostic value of the TSPAN4 expression as well as the immune analysis were investigated.

TSPANs, such as TSPAN4 and TSPAN7, as well as migrasomes play a critical role in vascular homeostasis (Zhang et al., 2020). Migrasome was demonstrated as an organelle to release cytoplasmic contents during cell migration and cholesterol and TSPAN4 are essential for migrasome formation (Ma et al., 2015; Yu, 2021). Migrating cells, such as monocytes and macrophages, could expel dysfunctional mitochondria in migrasomes to maintain mitochondrial homeostasis and control mitochondrial quality (Jiao et al., 2021; Mehra and Pernas, 2021; Sung et al., 2021). As a novel exosome-like organelle during cell migrations, lateral transfer of mRNA, protein, and even damaged mitochondria can modify the recipient cells (da Rocha-Azevedo and Schmid, 2015; Zhu et al., 2021; Baumann, 2021). The infiltration abundance of migrating cells, such as monocytes and macrophages, may explain the expression levels of TSPAN4 and its impact on prognosis are largely different among tumor types, which was consisted with the previous report by Huang et al., 2022. Huang et al. (2022) reported samples of different tumor types exhibited variable results in immune subtype analysis. Six immune subtypes were correlated tumors, and the expression of TSPAN4 was different among them (Huang et al., 2022).

Migrasomes take center stage in disease development (Tavano and Heisenberg, 2019) and TSPAN4 are essential for migrasome formation. TSPAN4 expression was highly expressed in atherosclerosis and can also be prognostic biomarkers in patients with ACC, GBM, LGG, LUSC, MESO, PCPG, PRAD, THYM, UVM, THCA, GBMLGG or LUADLUSC, while TSPAN4 expression can be diagnostic biomarkers in patients with LUSC, UCS, THYM, KIRC, PAAD, OV, DLBC, CESC and UCEC (AUC > .9) as well as LUAD, LIHC, PRAD, KICH, ESCA, BLCA, GBM, KIRP, BRCA, READ and COADREAD (AUC > .8). Deng et al. (2021) reported that TSPAN4 expression increased, enhancing proliferation and invasion in gastric cancer tissues (Qi et al., 2018). High TSPAN4 and ELAVL2 expression levels were independent risk factors for poor chemotherapy response in ESCC patients (Zhao et al., 2019). A TSPAN4-CD151 fusion gene was highly expressed in a pediatric infratentorial anaplastic ependymoma (Olsen et al., 2016). In addition, TSPAN4 was one of the top hub genes in patients with fulminant type 1 diabetes (Ye et al., 2020). In this study, TSPAN4 expression was correlated to the abundance of macrophages and DC cells in GBM and GBMLGG and we still need to investigate the functions of TSPAN4 expression on patients with atherosclerosis and cancer progression.

This study provided a critical role of TSPAN4 aberrant expression in the progression of atherosclerosis and pan-cancer. The quality of collected data relies on the source, which could show an impact on the conclusion. Secondly, the result and conclusions are not experimentally validated in the laboratory or clinic. Further studies are still needed to validate *in vivo* and *in vitro*.

## 4 Conclusion

TSPAN4 is differentially expressed in atherosclerosis and also in pan-cancer, which was associated with the progression and immune cell infiltration of the tumor, especially in GBM and GBMLGG. Therefore, TSPAN4 may serve as a potential prognostic and diagnostic biomarker and the crosstalk between atherosclerosis and tumor progression.

## Data Availability

Publicly available datasets were analyzed in this study. This data can be found here: The data can be downloaded from GEO datasets and TCGA database.

## Glossary

ACC: Adrenocortical carcinoma
AS: Atherosclerosis
AUC: Area under curve
BLCA: Bladder Urothelial Carcinoma
BRCA: Breast invasive carcinoma
CAD: Cardiovascular diseases
CESC: Cervical squamous cell carcinoma and endocervical adenocarcinoma
CHOL: Cholangiocarcinoma
CI: Confident interval
COAD: Colon adenocarcinoma
COADREAD: Colon adenocarcinoma and Rectum adenocarcinoma esophageal carcinoma
DEGs: Differentially expressed genes
DLBC: Lymphoid Neoplasm Diffuse Large B-cell Lymphoma
DSS: Disease specific survival
EPCAM: Epithelial cell adhesion molecule
ESCA: Esophageal carcinoma
GBM: Glioblastoma multiforme
GBMLGG: Glioblastoma multiforme and Brain Lower Grade Glioma
GO: Gene Ontology
GSEA: Gene Set Enrichment Analysis
GTEx: Genotype-Tissue Expression
HR: Hazard ratio
HNSC: Head and Neck squamous cell carcinoma
H4R: histamine H4 receptor
KEGG: Kyoto Encyclopedia of Genes and Genomes
KICH: Kidney Chromophobe
KIRC: Kidney renal clear cell carcinoma
KIRP: Kidney renal papillary cell carcinoma
LAML: Acute Myeloid Leukemia
LGG: Brain Lower Grade Glioma
LIHC: Liver hepatocellular carcinoma
LUAD: Lung adenocarcinoma
LUADLUSC: Lung adenocarcinoma and Lung squamous cell carcinoma
LUSC: Lung squamous cell carcinoma
MI: Myocardial infarction
MLH1: MutL homologous gene 1
MMR: Mismatch repair
MSH2: MutS homologous gene 2
MSH6: MutS homologous gene 6
NETs: Neutrophil extracellular traps
OS: Overall survival
OV: Ovarian serous cystadenocarcinoma
PAAD: Pancreatic adenocarcinoma
PFI: Progress free interval
PMS2: Increased separation after meiosis
PPI: Protein-protein interaction
PRAD: Prostate adenocarcinoma
READ: Rectum adenocarcinoma
ROC: Receiver operation characteristic
SKCM: Skin Cutaneous Melanoma
THCA: Thyroid carcinoma
THYM: Thymoma
TSPAN4: Tetraspanin 4
TSPAN7: Tetraspanin 7
UCEC: Uterine Corpus Endometrial Carcinoma
UCS: Uveal Melanoma

## References

[B1] Ashburner M. Ball C. A. Blake J. A. Botstein D. Butler H. Cherry J. M. (2000). Gene ontology: Tool for the unification of biology. The gene Ontology consortium. Nat. Genet. 25 (1), 25–29. 10.1038/75556 10802651 PMC3037419

[B2] Balzan S. Lubrano V. (2018). LOX-1 receptor: A potential link in atherosclerosis and cancer. Life Sci. 198, 79–86. 10.1016/j.lfs.2018.02.024 29462603

[B3] Baumann K. (2021). Damaged mitochondria are discarded via migrasomes. Nat. Rev. Mol. Cell Biol. 22 (7), 442. 10.1038/s41580-021-00388-0 34108688

[B4] Chang J. Liu X. Sun Y. (2017). Mortality due to acutemyocardial infarction in China from 1987 to 2014: Secular trends and ageperiod-cohort effects. Int. J. Cardiol. 227, 229–238. 10.1016/j.ijcard.2016.11.130 27839815

[B5] da Rocha-Azevedo B. Schmid S. L. (2015). Migrasomes: A new organelle of migrating cells. Cell Res. 25 (1), 1–2. 10.1038/cr.2014.146 25378181 PMC4650585

[B6] Deng Y. Cai S. Shen J. Peng H. (2021). Tetraspanins: Novel molecular regulators of gastric cancer. Front. Oncol. 11, 702510. 10.3389/fonc.2021.702510 34222025 PMC8250138

[B7] Dungal N. Benediktsson T. (1958). Gastric cancer and atherosclerosis. Lancet 1 (7027), 931–932. 10.1016/s0140-6736(58)91685-4 13540216

[B8] Dutta P. Courties G. Wei Y. Leuschner F. Gorbatov R. Robbins C. S. (2012). Myocardial infarction accelerates atherosclerosis. Nature 487, 325–329. 10.1038/nature11260 22763456 PMC3401326

[B9] Hägg D. A. Jernås M. Wiklund O. Thelle D. S. Fagerberg B. Eriksson P. (2008). Expression profiling of macrophages from subjects with atherosclerosis to identify novel susceptibility genes. Int. J. Mol. Med. 21 (6), 697–704. 10.3892/ijmm.21.6.697 18506362

[B10] Huang R. Sun H. Lin R. Zhang J. Yin H. Xian S. (2022). The role of tetraspanins pan-cancer. iScience 25 (8), 104777. 10.1016/j.isci.2022.104777 35992081 PMC9385710

[B11] Huang Y. Zucker B. Zhang S. Elias S. Zhu Y. Chen H. (2019). Migrasome formation is mediated by assembly of micron-scale tetraspanin macrodomains. Nat. Cell Biol. 21 (8), 991–1002. 10.1038/s41556-019-0367-5 31371828

[B12] Jiang D. Jiang Z. Lu D. Wang X. Liang H. Zhang J. (2019). Migrasomes provide regional cues for organ morphogenesis during zebrafish gastrulation. Nat. Cell Biol. 21 (8), 966–977. 10.1038/s41556-019-0358-6 31371827

[B13] Jiao H. Jiang D. Hu X. Du W. Ji L. Yang Y. (2021). Mitocytosis, a migrasome-mediated mitochondrial quality-control process. Cell 184 (11), 2896–2910.e13. e13. 10.1016/j.cell.2021.04.027 34048705

[B14] Kanehisa M. Goto S. (2000). Kegg: Kyoto encyclopedia of genes and genomes. Nucleic Acids Res. 28 (1), 27–30. 10.1093/nar/28.1.27 10592173 PMC102409

[B15] Koelwyn G. J. Newman A. A. C. Afonso M. S. van Solingen C. Corr E. M. Brown E. J. (2020). Myocardial infarction accelerates breast cancer via innate immune reprogramming. Nat. Med. 26 (9), 1452–1458. 10.1038/s41591-020-0964-7 32661390 PMC7789095

[B16] Liu Y. Li S. Rong W. Zeng C. Zhu X. Chen Q. (2020). Podocyte-released migrasomes in urine serve as an indicator for early podocyte injury. Kidney Dis. (Basel) 6 (6), 422–433. 10.1159/000511504 33313063 PMC7706499

[B17] Ma L. Li Y. Peng J. Wu D. Zhao X. Cui Y. (2015). Discovery of the migrasome, an organelle mediating release of cytoplasmic contents during cell migration. Cell Res. 25 (1), 24–38. 10.1038/cr.2014.135 25342562 PMC4650581

[B18] Ma X. Verweij E. W. E. Siderius M. Leurs R. Vischer H. F. (2021). Identification of TSPAN4 as novel histamine H(4) receptor interactor. Biomolecules 11 (8), 1127. 10.3390/biom11081127 34439793 PMC8394291

[B19] Mehra C. Pernas L. (2021). Move it to lose it: Mitocytosis expels damaged mitochondria. Dev. Cell 56 (14), 2014–2015. 10.1016/j.devcel.2021.07.001 34314697

[B20] Mensah S. A. Nersesyan A. A. Ebong E. E. (2021). Endothelial glycocalyx-mediated intercellular interactions: Mechanisms and implications for atherosclerosis and cancer metastasis. Cardiovasc Eng. Technol. 12 (1), 72–90. 10.1007/s13239-020-00487-7 33000443 PMC7904750

[B21] Moschonas I. C. Tselepis A. D. (2019). The pathway of neutrophil extracellular traps towards atherosclerosis and thrombosis. Atherosclerosis 288, 9–16. 10.1016/j.atherosclerosis.2019.06.919 31280097

[B22] Mukai M. Komori K. Oka T. (2018). Mechanism and management of cancer chemotherapy-induced atherosclerosis. J. Atheroscler. Thromb. 25 (10), 994–1002. 10.5551/jat.RV17027 30224607 PMC6193189

[B23] Naghavi M. Abajobir A. A. Abbafati C. Abbas K. M. Abd-Allah F. Abera S. F. (2017). Global, regional, and national age-sex specific mortality for 264 causes of death, 1980–2016: A systematic analysis for the global burden of disease study 2016. Lancet 390, 1151–1210. 10.1016/S0140-6736(17)32152-9 28919116 PMC5605883

[B24] Olsen T. K. Panagopoulos I. Gorunova L. Micci F. Andersen K. Kilen Andersen H. (2016). Novel fusion genes and chimeric transcripts in ependymal tumors. Genes Chromosom. Cancer 55 (12), 944–953. 10.1002/gcc.22392 27401149

[B25] Padgett L. E. Araujo D. J. Hedrick C. C. Olingy C. E. (2020). Functional crosstalk between T cells and monocytes in cancer and atherosclerosis. J. Leukoc. Biol. 108 (1), 297–308. 10.1002/JLB.1MIR0420-076R 32531833 PMC8006924

[B26] Qi W. Sun L. Liu N. Zhao S. Lv J. Qiu W. (2018). Tetraspanin family identified as the central genes detected in gastric cancer using bioinformatics analysis. Mol. Med. Rep. 18 (4), 3599–3610. 10.3892/mmr.2018.9360 30106120 PMC6131613

[B27] Restall I. J. Cseh O. Richards L. M. Pugh T. J. Luchman H. A. Weiss S. (2020). Brain tumor stem cell dependence on glutaminase reveals a metabolic vulnerability through the amino acid deprivation response pathway. Cancer Res. 80 (24), 5478–5490. 10.1158/0008-5472.CAN-19-3923 33106333

[B28] Shannon P. Markiel A. Ozier O. Baliga N. S. Wang J. T. Ramage D. (2003). Cytoscape: A software environment for integrated models of biomolecular interaction networks. Genome Res. 13 (11), 2498–2504. 10.1101/gr.1239303 14597658 PMC403769

[B29] Singh A. Gupta A. DeFilippis E. M. Qamar A. Biery D. W. Almarzooq Z. (2020). Cardiovascular mortality after type 1 and type 2 myocardial infarction in young adults. J. Am. Coll. Cardiol. 75 (9), 1003–1013. 10.1016/j.jacc.2019.12.052 32138959 PMC7382936

[B30] Subramanian A. Tamayo P. Mootha V. K. Mukherjee S. Ebert B. L. Gillette M. A. (2005). Gene set enrichment analysis: A knowledge-based approach for interpreting genome-wide expression profiles. Proc. Natl. Acad. Sci. U. S. A. 102 (43), 15545–15550. 10.1073/pnas.0506580102 16199517 PMC1239896

[B31] Subramanian A. Tamayo P. Mootha V. K. Mukherjee S. Ebert B. L. Gillette M. A. (2005). Gene set enrichment analysis: A knowledge-based approach for interpreting genome-wide expression profiles. Proc. Natl. Acad. Sci. U. S. A. 102 (43), 15545–15550. 10.1073/pnas.0506580102 16199517 PMC1239896

[B32] Sung B. H. Parent C. A. Weaver A. M. (2021). Extracellular vesicles: Critical players during cell migration. Dev. Cell 56 (13), 1861–1874. 10.1016/j.devcel.2021.03.020 33811804 PMC8282723

[B33] Szklarczyk D. Gable A. L. Lyon D. Junge A. Wyder S. Huerta-Cepas J. (2019). STRING v11: Protein-protein association networks with increased coverage, supporting functional discovery in genome-wide experimental datasets. Nucleic Acids Res. 47 (D1), D607–D613. 10.1093/nar/gky1131 30476243 PMC6323986

[B34] Tang Z. Li C. Kang B. Gao G. Li C. Zhang Z. (2017). Gepia: A web server for cancer and normal gene expression profiling and interactive analyses. Nucleic Acids Res. 45 (W1), W98–W102. 10.1093/nar/gkx247 28407145 PMC5570223

[B35] Tapia-Vieyra J. V. Delgado-Coello B. Mas-Oliva J. (2017). Atherosclerosis and cancer; A resemblance with far-reaching implications. Arch. Med. Res. 48 (1), 12–26. 10.1016/j.arcmed.2017.03.005 28577865

[B36] Tavano S. Heisenberg C. P. (2019). Migrasomes take center stage. Nat. Cell Biol. 21 (8), 918–920. 10.1038/s41556-019-0369-3 31371826

[B37] Yang L. Liu Q. Zhang X. Liu X. Zhou B. Chen J. (2020). DNA of neutrophil extracellular traps promotes cancer metastasis via CCDC25. Nature 583 (7814), 133–138. 10.1038/s41586-020-2394-6 32528174

[B38] Ye X. Zeng T. Kong W. Chen L. L. (2020). Integrative analyses of genes associated with fulminant type 1 diabetes. J. Immunol. Res. 2020, 1025857. 10.1155/2020/1025857 33083497 PMC7559223

[B39] Yu G. C. Wang L. G. Han Y. Y. He Q. Y. (2012). clusterProfiler: an R package for comparing biological themes among gene clusters. Omics a J. Integr. Biol. 16 (5), 284–287. 10.1089/omi.2011.0118 PMC3339379 22455463

[B40] Yu L. (2021). Migrasomes: The knowns, the known unknowns and the unknown unknowns: A personal perspective. Sci. China Life Sci. 64 (1), 162–166. 10.1007/s11427-020-1827-8 33190174

[B41] Zhang Y. Wang J. Ding Y. Zhang J. Xu Y. Xu J. (2020). Migrasome and tetraspanins in vascular homeostasis: Concept, present, and future. Front. Cell Dev. Biol. 8, 438. 10.3389/fcell.2020.00438 32612990 PMC7308473

[B42] Zhao W. S. Yan W. P. Chen D. B. Dai L. Yang Y. B. Kang X. Z. (2019). Genome-scale CRISPR activation screening identifies a role of ELAVL2-CDKN1A axis in paclitaxel resistance in esophageal squamous cell carcinoma. Am. J. Cancer Res. 9 (6), 1183–1200. 31285951 PMC6610048

[B43] Zhu M. Zou Q. Huang R. Li Y. Xing X. Fang J. (2021). Lateral transfer of mRNA and protein by migrasomes modifies the recipient cells. Cell Res. 31 (2), 237–240. 10.1038/s41422-020-00415-3 32994478 PMC8026638

