# Supplementary figures and images for: TSPAN4 is a prognostic and immune target in Glioblastoma multiforme
Source: Front Mol Biosci. 2023 Jan 6;9:1030057. doi: 10.3389/fmolb.2022.1030057 (PMC9853066; doi:10.3389/fmolb.2022.1030057)

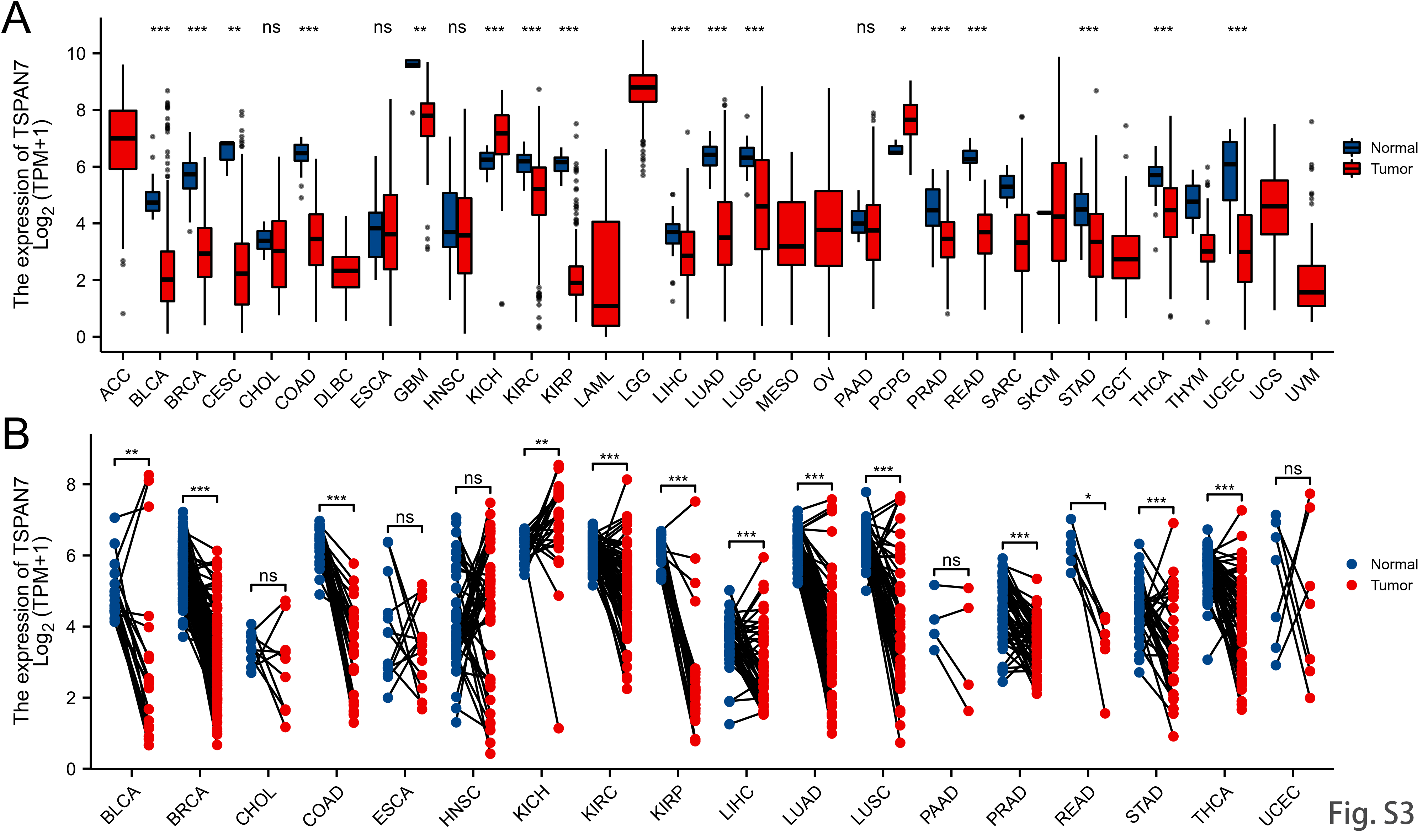

Supplement: Supplementary file 1 [file Image3.TIFF]

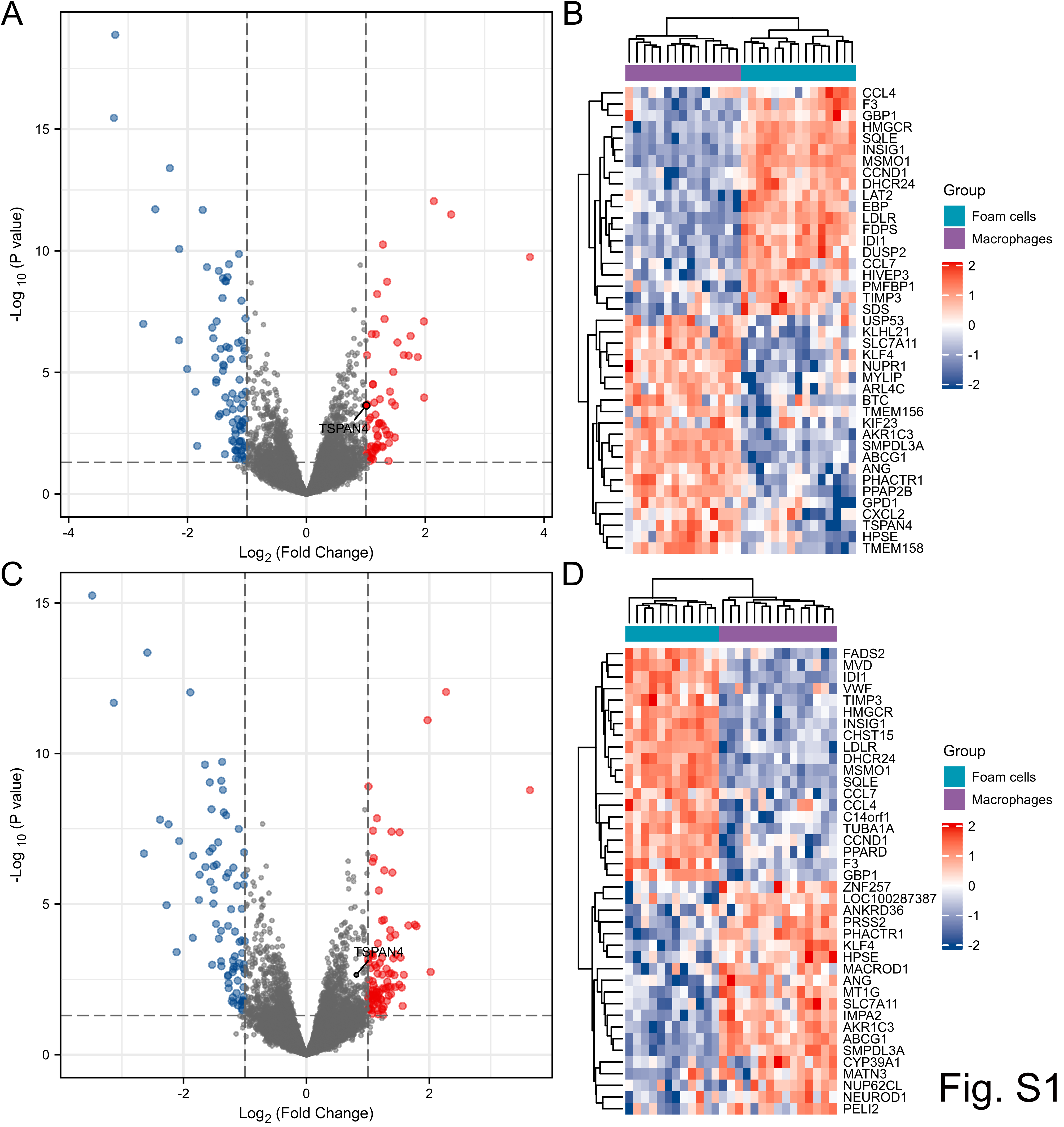

Supplement: Supplementary file 2 [file Image1.TIFF]

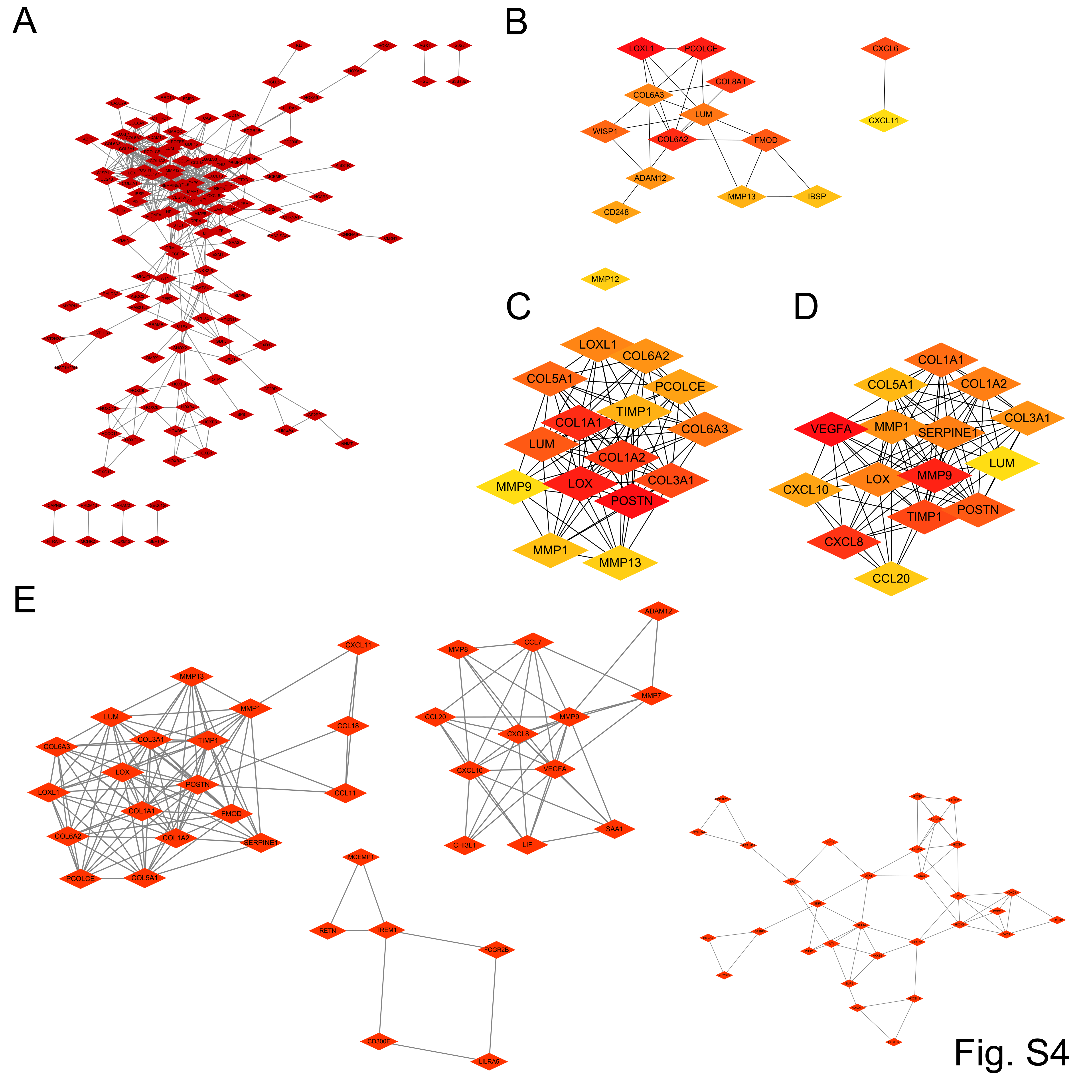

Supplement: Supplementary file 4 [file Image4.TIF]

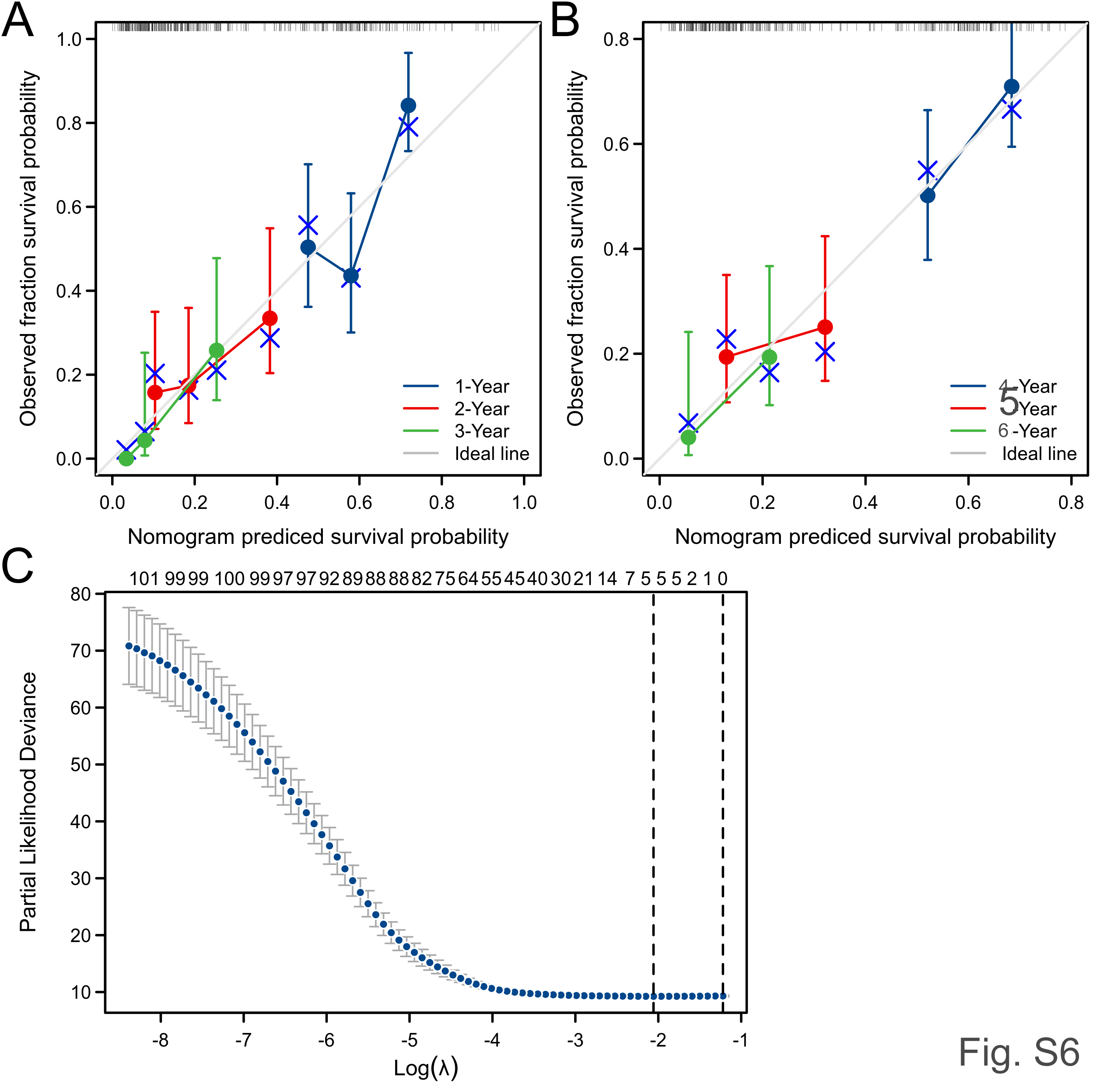

Supplement: Supplementary file 5 [file Image6.TIFF]

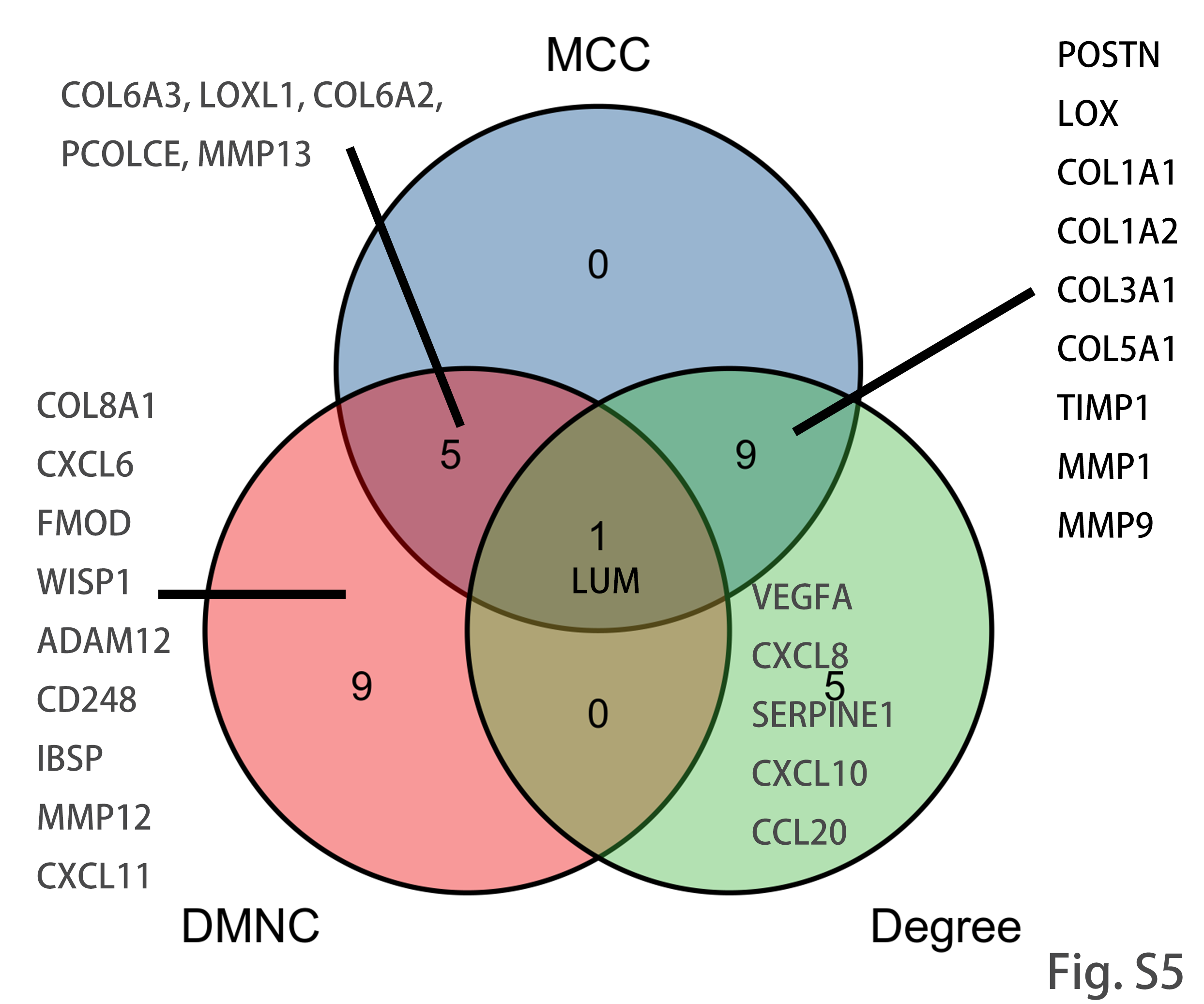

Supplement: Supplementary file 6 [file Image5.TIF]

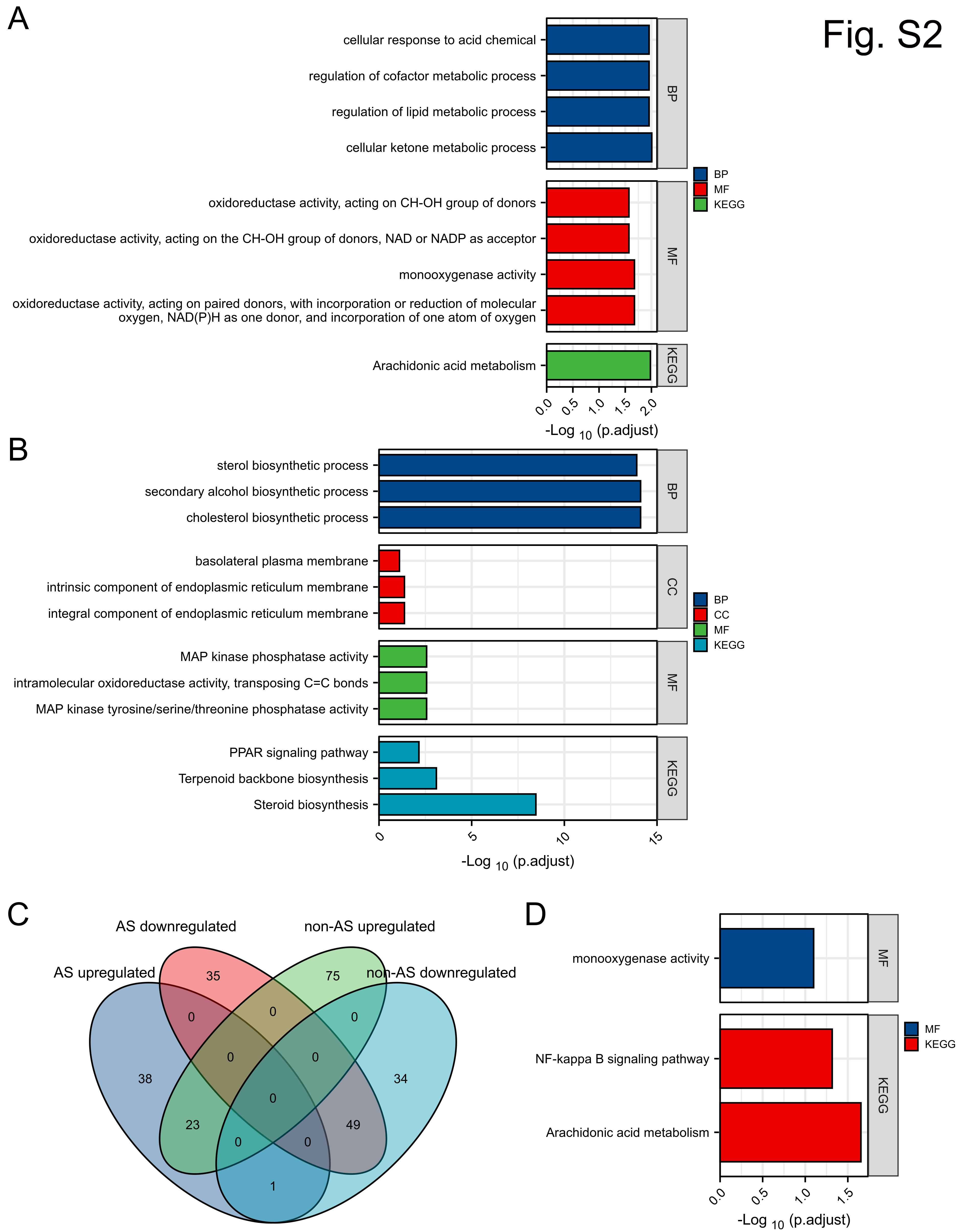

Supplement: Supplementary file 7 [file Image2.TIFF]

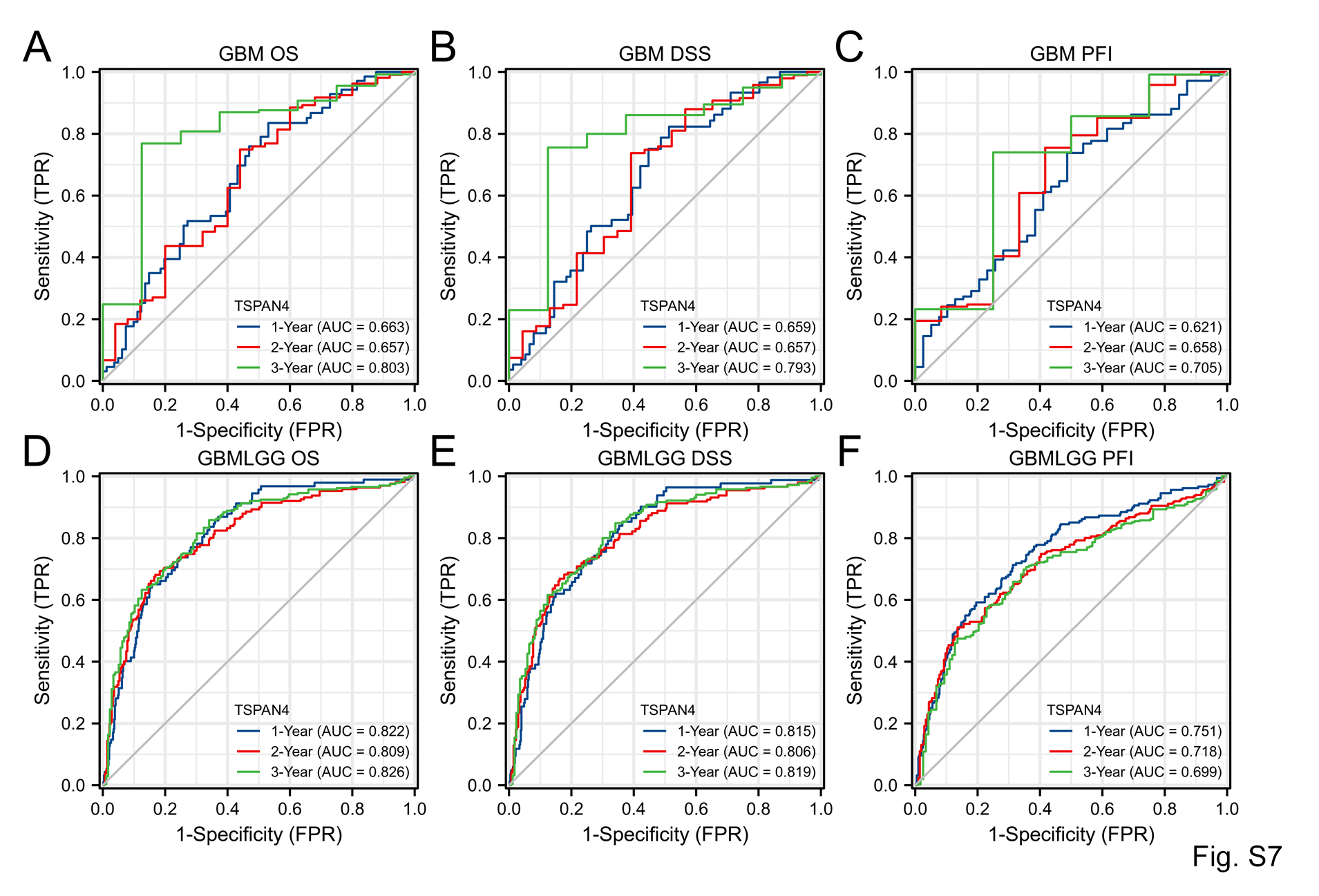

Supplement: Supplementary file 8 [file Image7.TIFF]
