# Supplementary material for: TSPAN4 is a prognostic and immune target in Glioblastoma multiforme
Source: Front Mol Biosci. 2023 Jan 6;9:1030057. doi: 10.3389/fmolb.2022.1030057 (PMC9853066; doi:10.3389/fmolb.2022.1030057)
Supplement: Supplementary file 3 [file Table1.DOCX]

Supplementary Table 1. The GO/KEGG pathways enriched by up-regulated DEGs in foam cells from subject with atherosclerosis.

| ONTOLOGY | ID | Description | GeneRatio | BgRatio | pvalue | p.adjust | qvalue |
| --- | --- | --- | --- | --- | --- | --- | --- |
| BP | GO:0042180 | cellular ketone metabolic process | 7/52 | 248/18670 | 5.39e-06 | 0.010 | 0.008 |
| BP | GO:0019216 | regulation of lipid metabolic process | 8/52 | 410/18670 | 1.63e-05 | 0.011 | 0.009 |
| BP | GO:0051193 | regulation of cofactor metabolic process | 5/52 | 119/18670 | 1.98e-05 | 0.011 | 0.009 |
| BP | GO:0071229 | cellular response to acid chemical | 6/52 | 209/18670 | 2.43e-05 | 0.011 | 0.009 |
| BP | GO:0062012 | regulation of small molecule metabolic process | 8/52 | 459/18670 | 3.66e-05 | 0.013 | 0.010 |
| MF | GO:0016709 | oxidoreductase activity, acting on paired donors, with incorporation or reduction of molecular oxygen, NAD(P)H as one donor, and incorporation of one atom of oxygen | 3/52 | 39/17697 | 2.03e-04 | 0.021 | 0.017 |
| MF | GO:0004497 | monooxygenase activity | 4/52 | 99/17697 | 2.03e-04 | 0.021 | 0.017 |
| MF | GO:0016616 | oxidoreductase activity, acting on the CH-OH group of donors, NAD or NADP as acceptor | 4/52 | 119/17697 | 4.10e-04 | 0.027 | 0.021 |
| MF | GO:0016614 | oxidoreductase activity, acting on CH-OH group of donors | 4/52 | 128/17697 | 5.40e-04 | 0.027 | 0.021 |
| MF | GO:0004032 | alditol:NADP+ 1-oxidoreductase activity | 2/52 | 13/17697 | 6.47e-04 | 0.027 | 0.021 |
| KEGG | hsa00590 | Arachidonic acid metabolism | 4/32 | 63/8076 | 1.03e-04 | 0.010 | 0.010 |

DEGs, Different Expressed Genes; GO, Gene ONTOLOGY; BP, Biological Process; CC, cellular component; MF, Molecular Function; KEGG, Kyoto Encyclopedia of Genes and Genomes.

Supplementary Table 2. The GO/KEGG pathways enriched by down-regulated DEGs in foam cells from subject with atherosclerosis.

| ONTOLOGY | ID | Description | GeneRatio | BgRatio | pvalue | p.adjust | qvalue |
| --- | --- | --- | --- | --- | --- | --- | --- |
| BP | GO:0006695 | cholesterol biosynthetic process | 13/80 | 75/18670 | 6.06e-18 | 7.72e-15 | 6.42e-15 |
| BP | GO:1902653 | secondary alcohol biosynthetic process | 13/80 | 76/18670 | 7.28e-18 | 7.72e-15 | 6.42e-15 |
| BP | GO:0016126 | sterol biosynthetic process | 13/80 | 81/18670 | 1.76e-17 | 1.24e-14 | 1.03e-14 |
| BP | GO:0008203 | cholesterol metabolic process | 15/80 | 150/18670 | 7.80e-17 | 4.14e-14 | 3.44e-14 |
| BP | GO:1902652 | secondary alcohol metabolic process | 15/80 | 155/18670 | 1.28e-16 | 5.45e-14 | 4.53e-14 |
| CC | GO:0030176 | integral component of endoplasmic reticulum membrane | 5/81 | 150/19717 | 3.84e-04 | 0.043 | 0.038 |
| CC | GO:0031227 | intrinsic component of endoplasmic reticulum membrane | 5/81 | 158/19717 | 4.87e-04 | 0.043 | 0.038 |
| CC | GO:0016323 | basolateral plasma membrane | 5/81 | 217/19717 | 0.002 | 0.079 | 0.071 |
| CC | GO:0005777 | peroxisome | 4/81 | 134/19717 | 0.002 | 0.079 | 0.071 |
| CC | GO:0042579 | microbody | 4/81 | 134/19717 | 0.002 | 0.079 | 0.071 |
| MF | GO:0017017 | MAP kinase tyrosine/serine/threonine phosphatase activity | 3/78 | 13/17697 | 2.28e-05 | 0.003 | 0.002 |
| MF | GO:0016863 | intramolecular oxidoreductase activity, transposing C=C bonds | 3/78 | 14/17697 | 2.90e-05 | 0.003 | 0.002 |
| MF | GO:0033549 | MAP kinase phosphatase activity | 3/78 | 15/17697 | 3.61e-05 | 0.003 | 0.002 |
| MF | GO:0016627 | oxidoreductase activity, acting on the CH-CH group of donors | 4/78 | 58/17697 | 1.24e-04 | 0.007 | 0.006 |
| MF | GO:0016628 | oxidoreductase activity, acting on the CH-CH group of donors, NAD or NADP as acceptor | 3/78 | 26/17697 | 1.99e-04 | 0.009 | 0.007 |
| KEGG | hsa00100 | Steroid biosynthesis | 7/52 | 20/8076 | 2.19e-11 | 3.45e-09 | 3.26e-09 |
| KEGG | hsa00900 | Terpenoid backbone biosynthesis | 4/52 | 22/8076 | 1.03e-05 | 8.06e-04 | 7.62e-04 |
| KEGG | hsa03320 | PPAR signaling pathway | 5/52 | 78/8076 | 1.35e-04 | 0.007 | 0.007 |
| KEGG | hsa04010 | MAPK signaling pathway | 8/52 | 294/8076 | 5.23e-04 | 0.021 | 0.019 |
| KEGG | hsa05221 | Acute myeloid leukemia | 4/52 | 67/8076 | 8.68e-04 | 0.027 | 0.026 |

DEGs, Different Expressed Genes; GO, Gene ONTOLOGY; BP, Biological Process; CC, cellular component; MF, Molecular Function; KEGG, Kyoto Encyclopedia of Genes and Genomes.

Supplementary Table 3. The GO/KEGG pathways enriched by 39 up-regulated DEGs (AS upregulated not non-AS upregulated DEGs).

| ONTOLOGY | ID | Description | GeneRatio | BgRatio | pvalue | p.adjust | qvalue |
| --- | --- | --- | --- | --- | --- | --- | --- |
| MF | GO:0004497 | monooxygenase activity | 3/30 | 99/17697 | 6.18e-04 | 0.080 | 0.059 |
| KEGG | hsa00590 | Arachidonic acid metabolism | 3/17 | 63/8076 | 2.85e-04 | 0.022 | 0.022 |
| KEGG | hsa04064 | NF-kappa B signaling pathway | 3/17 | 104/8076 | 0.001 | 0.048 | 0.048 |

DEGs, Different Expressed Genes; GO, Gene ONTOLOGY; MF, Molecular Function; KEGG, Kyoto Encyclopedia of Genes and Genomes.

Supplementary Table 4. The GO/KEGG pathways enriched by top 50 co-expression genes positively related to TSPAN4 expression in GBMLGG.

| ONTOLOGY | ID | Description | GeneRatio | BgRatio | pvalue | p.adjust | qvalue |
| --- | --- | --- | --- | --- | --- | --- | --- |
| BP | GO:0043062 | extracellular structure organization | 14/49 | 422/18670 | 2.41e-12 | 2.86e-09 | 2.30e-09 |
| BP | GO:0030198 | extracellular matrix organization | 12/49 | 368/18670 | 1.38e-10 | 8.16e-08 | 6.57e-08 |
| BP | GO:0035966 | response to topologically incorrect protein | 8/49 | 199/18670 | 4.49e-08 | 1.41e-05 | 1.14e-05 |
| BP | GO:0034976 | response to endoplasmic reticulum stress | 9/49 | 285/18670 | 4.78e-08 | 1.41e-05 | 1.14e-05 |
| BP | GO:0030199 | collagen fibril organization | 5/49 | 54/18670 | 2.90e-07 | 6.87e-05 | 5.53e-05 |
| CC | GO:0005788 | endoplasmic reticulum lumen | 16/50 | 309/19717 | 2.74e-17 | 3.04e-15 | 1.61e-15 |
| CC | GO:0062023 | collagen-containing extracellular matrix | 14/50 | 406/19717 | 9.43e-13 | 5.23e-11 | 2.78e-11 |
| CC | GO:0098644 | complex of collagen trimers | 4/50 | 19/19717 | 1.38e-07 | 5.10e-06 | 2.71e-06 |
| CC | GO:0005793 | endoplasmic reticulum-Golgi intermediate compartment | 6/50 | 126/19717 | 7.63e-07 | 2.12e-05 | 1.12e-05 |
| CC | GO:0005583 | fibrillar collagen trimer | 3/50 | 11/19717 | 2.50e-06 | 4.28e-05 | 2.27e-05 |
| MF | GO:0030020 | extracellular matrix structural constituent conferring tensile strength | 5/50 | 41/17697 | 1.02e-07 | 9.24e-06 | 6.03e-06 |
| MF | GO:0019838 | growth factor binding | 7/50 | 137/17697 | 1.08e-07 | 9.24e-06 | 6.03e-06 |
| MF | GO:0005201 | extracellular matrix structural constituent | 7/50 | 163/17697 | 3.54e-07 | 2.02e-05 | 1.32e-05 |
| MF | GO:0048407 | platelet-derived growth factor binding | 3/50 | 11/17697 | 3.45e-06 | 1.47e-04 | 9.61e-05 |
| MF | GO:0005178 | integrin binding | 5/50 | 132/17697 | 3.46e-05 | 0.001 | 7.73e-04 |
| KEGG | hsa04974 | Protein digestion and absorption | 5/28 | 103/8076 | 2.38e-05 | 0.002 | 0.002 |
| KEGG | hsa00531 | Glycosaminoglycan degradation | 3/28 | 19/8076 | 3.49e-05 | 0.002 | 0.002 |
| KEGG | hsa04512 | ECM-receptor interaction | 4/28 | 88/8076 | 2.21e-04 | 0.006 | 0.006 |
| KEGG | hsa04141 | Protein processing in endoplasmic reticulum | 5/28 | 171/8076 | 2.66e-04 | 0.006 | 0.006 |
| KEGG | hsa04933 | AGE-RAGE signaling pathway in diabetic complications | 4/28 | 100/8076 | 3.61e-04 | 0.007 | 0.006 |

DEGs, Different Expressed Genes; GO, Gene ONTOLOGY; BP, Biological Process; CC, cellular component; MF, Molecular Function; KEGG, Kyoto Encyclopedia of Genes and Genomes.

Supplementary Table 5. The GO/KEGG pathways enriched by top 50 co-expression genes negatively related to TSPAN4 expression in GBMLGG.

| ONTOLOGY | ID | Description | GeneRatio | BgRatio | pvalue | p.adjust | qvalue |
| --- | --- | --- | --- | --- | --- | --- | --- |
| BP | GO:1990416 | cellular response to brain-derived neurotrophic factor stimulus | 3/48 | 10/18670 | 1.89e-06 | 0.001 | 0.001 |
| BP | GO:0090042 | tubulin deacetylation | 3/48 | 11/18670 | 2.59e-06 | 0.001 | 0.001 |
| BP | GO:0071804 | cellular potassium ion transport | 6/48 | 217/18670 | 1.88e-05 | 0.005 | 0.004 |
| BP | GO:0071805 | potassium ion transmembrane transport | 6/48 | 217/18670 | 1.88e-05 | 0.005 | 0.004 |
| BP | GO:0006813 | potassium ion transport | 6/48 | 240/18670 | 3.31e-05 | 0.007 | 0.006 |
| CC | GO:0097060 | synaptic membrane | 8/47 | 432/19717 | 7.40e-06 | 7.44e-04 | 4.98e-04 |
| CC | GO:0045211 | postsynaptic membrane | 7/47 | 323/19717 | 1.06e-05 | 7.44e-04 | 4.98e-04 |
| CC | GO:0098978 | glutamatergic synapse | 6/47 | 349/19717 | 1.72e-04 | 0.006 | 0.004 |
| CC | GO:0098984 | neuron to neuron synapse | 6/47 | 350/19717 | 1.74e-04 | 0.006 | 0.004 |
| CC | GO:0033267 | axon part | 6/47 | 382/19717 | 2.79e-04 | 0.008 | 0.005 |
| MF | GO:0099186 | structural constituent of postsynapse | 2/43 | 11/17697 | 3.13e-04 | 0.057 | 0.042 |
| MF | GO:0000900 | translation repressor activity, mRNA regulatory element binding | 2/43 | 15/17697 | 5.93e-04 | 0.057 | 0.042 |
| MF | GO:0098918 | structural constituent of synapse | 2/43 | 17/17697 | 7.66e-04 | 0.057 | 0.042 |
| MF | GO:0030371 | translation repressor activity | 2/43 | 26/17697 | 0.002 | 0.077 | 0.056 |
| MF | GO:0090079 | translation regulator activity, nucleic acid binding | 2/43 | 28/17697 | 0.002 | 0.077 | 0.056 |

DEGs, Different Expressed Genes; GO, Gene ONTOLOGY; BP, Biological Process; CC, cellular component; MF, Molecular Function.

Supplementary Table 6. The characteristic information of TSPAN4 low ang high groups in GBM.

| Characteristic | Low expression of TSPAN4 | High expression of TSPAN4 | p |
| --- | --- | --- | --- |
| n | 348 | 348 |  |
| WHO grade, n (%) |  |  | < 0.001 |
| G2 | 166 (26.1%) | 58 (9.1%) |  |
| G3 | 128 (20.2%) | 115 (18.1%) |  |
| G4 | 14 (2.2%) | 154 (24.3%) |  |
| IDH status, n (%) |  |  | < 0.001 |
| WT | 38 (5.5%) | 208 (30.3%) |  |
| Mut | 306 (44.6%) | 134 (19.5%) |  |
| 1p/19q codeletion, n (%) |  |  | 0.243 |
| codel | 79 (11.5%) | 92 (13.4%) |  |
| non-codel | 268 (38.9%) | 250 (36.3%) |  |
| Age, meidan (IQR) | 38 (31, 48) | 54 (42, 63) | < 0.001 |

Supplementary Table 7. The univariate analysis and multivariate analysis of patients’ characteristics.

| Characteristics | Total(N) | Univariate analysis | |  | Multivariate analysis | |
| --- | --- | --- | --- | --- | --- | --- |
|  |  | Hazard ratio (95% CI) | P value |  | Hazard ratio (95% CI) | P value |
| WHO grade | 634 |  |  |  |  |  |
| G2 | 223 | Reference |  |  |  |  |
| G3 | 243 | 2.999 (2.007-4.480) | **<0.001** |  | 2.117 (1.306-3.432) | **0.002** |
| G4 | 168 | 18.615 (12.460-27.812) | **<0.001** |  | 7.708 (2.340-25.386) | **<0.001** |
| 1p/19q codeletion | 688 |  |  |  |  |  |
| codel | 170 | Reference |  |  |  |  |
| non-codel | 518 | 4.428 (2.885-6.799) | **<0.001** |  | 1.033 (0.539-1.979) | 0.923 |
| Primary therapy outcome | 461 |  |  |  |  |  |
| PD | 112 | Reference |  |  |  |  |
| SD | 147 | 0.440 (0.294-0.658) | **<0.001** |  | 0.330 (0.198-0.547) | **<0.001** |
| PR | 64 | 0.170 (0.074-0.391) | **<0.001** |  | 0.193 (0.068-0.544) | **0.002** |
| CR | 138 | 0.133 (0.064-0.278) | **<0.001** |  | 0.153 (0.070-0.331) | **<0.001** |
| TSPAN4 | 695 | 1.962 (1.747-2.203) | **<0.001** |  | 0.735 (0.536-1.008) | 0.056 |
| Age | 695 |  |  |  |  |  |
| <=60 | 552 | Reference |  |  |  |  |
| >60 | 143 | 4.668 (3.598-6.056) | **<0.001** |  | 3.757 (2.267-6.226) | **<0.001** |
| IDH status | 685 |  |  |  |  |  |
| WT | 246 | Reference |  |  |  |  |
| Mut | 439 | 0.117 (0.090-0.152) | **<0.001** |  | 0.326 (0.178-0.595) | **<0.001** |
| Gender | 695 |  |  |  |  |  |
| Female | 297 | Reference |  |  |  |  |
| Male | 398 | 1.262 (0.988-1.610) | 0.062 |  | 1.693 (1.084-2.644) | **0.021** |
| Histological type | 695 |  |  |  |  |  |
| Astrocytoma | 195 | Reference |  |  |  |  |
| Glioblastoma | 168 | 6.791 (4.932-9.352) | **<0.001** |  |  |  |
| Oligoastrocytoma | 134 | 0.657 (0.419-1.031) | 0.068 |  | 1.286 (0.748-2.211) | 0.363 |
| Oligodendroglioma | 198 | 0.580 (0.395-0.853) | **0.006** |  | 0.773 (0.439-1.362) | 0.373 |
